# Synthesis and Biological Evaluation of Novel Water-Soluble Poly-(ethylene glycol)-10-hydroxycamptothecin Conjugates

**DOI:** 10.3390/molecules20059393

**Published:** 2015-05-21

**Authors:** Na Guo, Du Jiang, Luyao Wang, Xing You, Yu-Ou Teng, Peng Yu

**Affiliations:** Key Laboratory of Industrial Microbiology, Ministry of Education, Tianjin Key Laboratory of Industry Microbiology, College of Biotechnology, Tianjin University of Science and Technology, No.29, 13th Avenue, TEDA, Tianjin 300457, China; E-Mails: guona@tust.edu.cn (N.G.); jiangduwill@126.com (D.J.); wangluyao_1106@163.com (L.W.); youxing_2013@126.com (X.Y.); tyo201485@tust.edu.cn (Y.-O.T.)

**Keywords:** antitumor, PEG, hydroxycamptothecin, conjugate

## Abstract

In order to improve the antitumor activity and water solubility of 10-hydroxycamptothecin (HCPT), a series of novel HCPT conjugates were designed and synthesized by conjugating polyethylene glycol (PEG) to the 10-hydroxyl group of HCPT via a valine spacer. The *in vitro* stability of these synthesized compounds was determined in pH 7.4 buffer at 37 °C, and the results showed that they released HCPT at different rates. All the compounds demonstrated significant antitumor activity *in vitro* against K562, HepG2 and HT-29 cells. Among them, compounds, **4a**, **4d**, **4e** and **4f**, exhibited 2–5 times higher potency than HCPT. The stability and antitumor activity of these conjugates were found to be closely related to the length of PEG and the linker type, conjugates with a relatively short PEG chain and carbamate linkages (compounds **4a** and **4f**) exhibited controlled release of HCPT and excellent antitumor *in vitro* activity.

## 1. Introduction

Camptothecin (CPT) and 10-hydroxycamptothecin (HCPT) are natural occurred topoisomerase I inhibitors with strong antitumor activity both *in vitro* and *in vivo* [1,2]. However, the clinical use of these two compounds is limited by their low solubility in water and high toxicity. In recent years, the potential of synthetic macromolecules as carriers of anticancer drugs has attracted immense interest. Polymeric prodrugs of various cytotoxic agents with soluble polymers such as PEG have been extensively investigated. The polymeric conjugates could simultaneously act as both a transport form and a prodrug to enhance drug distribution in tumor sites by enhanced permeability and retention (EPR) effects and improved drug pharmacokinetic properties [3,4,5,6]. In the past few years, CPT has been covalently coupled at C-20 to various water-soluble polymers to improve its solubility [7], *i.e.*, PEG, poly(l-glutamic acid), β-cyclodextrin-based polymers, poly[*N*-(2-hydroxypropyl)-methacrylamide], poly[(*N*-carboxybutyl)-l-aspartamide] and poly(amido-amine).

**Figure 1:**
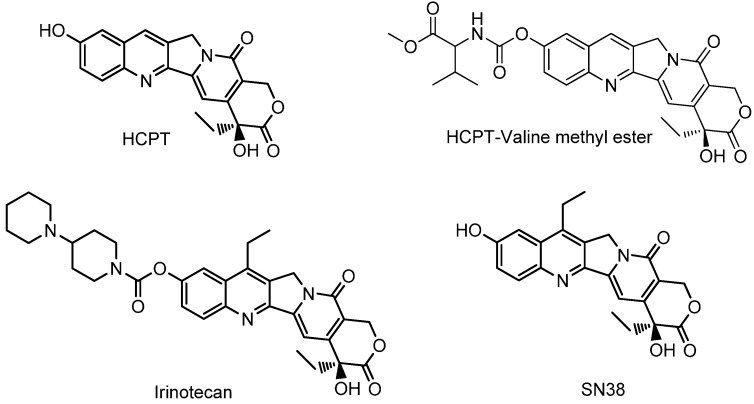
Structures of HCPT, HCPT-valine methyl ester, irinotecan and SN38.

PEG, a water-soluble, nontoxic, biodegradable and biocompatible polymer, is broadly used in medicine, food and cosmetics. Plenty of PEG-CPT conjugates have been reported, especially with an ester linkage [8]. However, PEG has seldom been applied for the synthesis of HCPT-PEG conjugates, especially with linkages other than ester ones, although HCPT is more active and less toxic than CPT. Derivation at C-10 of CPT analogues has led to some successful prodrugs, for example, irinotecan (Figure 1), which is a water-soluble prodrug of the CPT derivative SN-38 with a carbamate linkage. It is the most widely used CPT derivative for the treatment of cancer. However, the low bioconversion efficiency (4%–5%) from irinotecan to the active form SN-38 is responsible for individual variation in efficacy and toxicity [9,10,11].

In our previous study [12], a series of amino acid methyl ester conjugates of HCPT at C-10 were found to exhibit excellent antitumor activity and decreased toxicity against several normal cells, especially the valine methyl ester conjugated derivative (HCPT-Valine methyl ester, Figure 1). Here, we would like to report the design and synthesis of a series of PEG-HCPT conjugates based on this valine spacer. Our results demonstrated that the HCPT release and the antitumor activity are strongly dependent on the PEG length and the linker properties.

## 2. Results and Discussion

**Figure 2:**
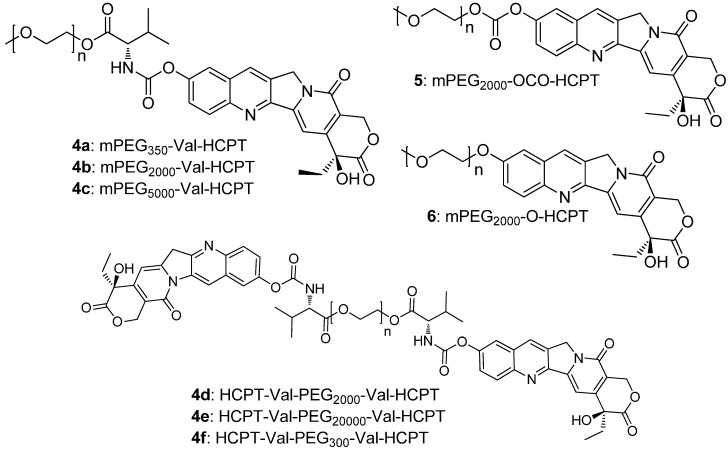
Structures of conjugates **4a**–**4f**, **5**, and **6**.

In order to conjugate PEG with HCPT, valine was chosen as a spacer to link the PEG and HCPT (compounds **4a**–**4f**). Meanwhile, PEG and HCPT were also conjugated directly via a carbonate group (compound **5**) or an ether group (compound **6**) as reference compounds to investigate the influence of the spacer on stability and activity (Figure 2).

### 2.1. Chemistry

**Scheme 1:**
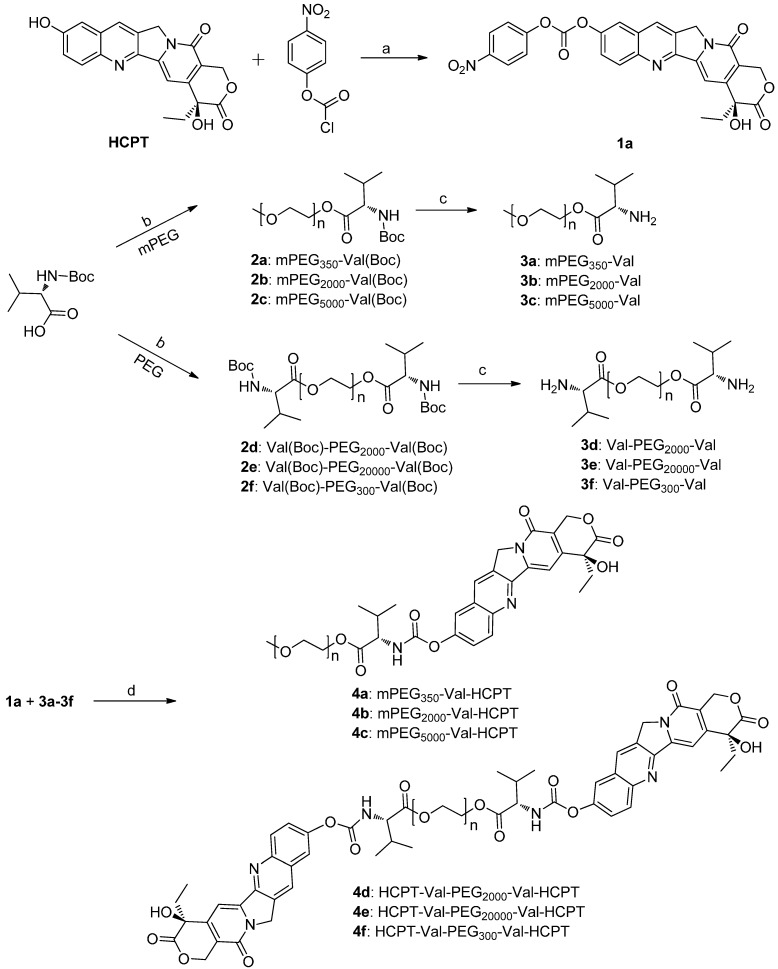
Synthesis of conjugates **4a**–**4f**.

The synthesis of the conjugates **4a**–**4f** is shown in Scheme 1. The key intermediate **1a** was synthesized in a good yield from HCPT and 4-nitrophenyl chloroformate in the presence of triethylamine. Meanwhile, Boc-valine was linked with monomethoxy-PEG (mPEG) or dihydroxy-PEG via an ester group to afford mPEG-valine-Boc (compounds **2a**–**2c**) or Boc-valine-PEG-valine-Boc (componds **2d**–**2f**), which were then deprotected with TFA and reacted with **1a** to provide the coupled compounds **4a**–**4f**.

**Scheme 2:**
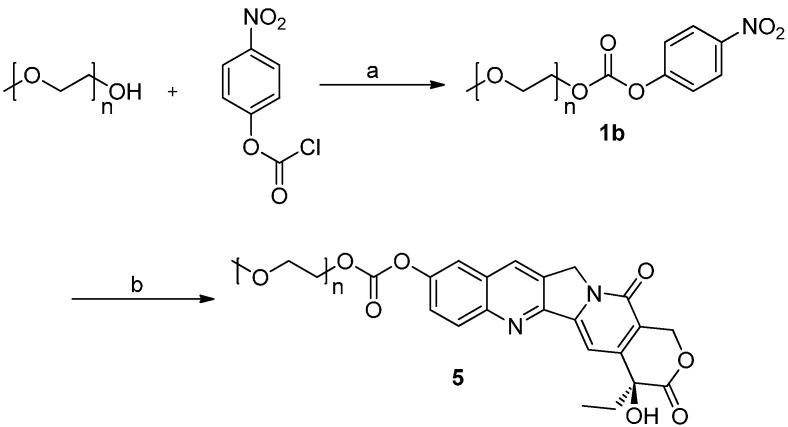
Synthesis of conjugate **5**.

**Scheme 3:**
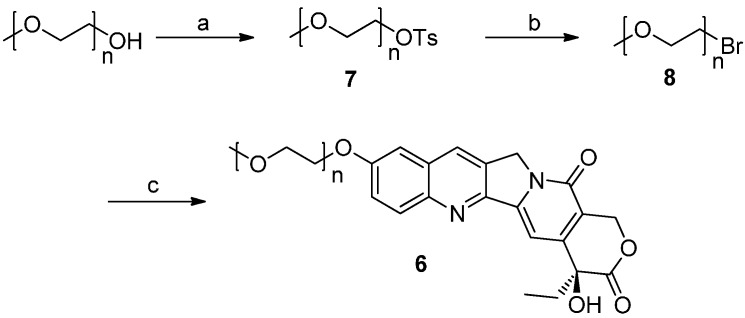
Synthesis of conjugate **6**.

Compound **5** (Scheme 2) was synthesized using a similar method to that described for compounds **4a**–**4f**. MPEG_2000_ was reacted with 4-nitrophenyl chloroformate to give the active intermediate **1b**, which was then reacted with HCPT to afford mPEG_2000_-OCO-HCPT (**5**). The synthesis of mPEG_2000_-O-HCPT (**6**) is shown in Scheme 3. MPEG_2000_ was converted into compound **8** via *p*-toluenesulfonate **7**. HCPT was reacted with **8** under basic conditions to afford compound **6** (Scheme 3).

*Reagents and conditions*: (a) Et_3_N, THF, rt. 1 h; (b) EDCI, DMAP, rt. overnight; (c) TFA, DCM, rt. 1 h; (d) DMF, DCM, rt. 1 h.

*Reagents and conditions*: (a) Et_3_N, DCM, rt. 3 h; (b) HCPT, Et_3_N, DMF, 60 °C, 45 min.

*Reagents and conditions*: (a) TsCl, Et_3_N, DCM, reflux, 5 h; (b) LiBr, acetone, reflux, overnight; (c) HCPT, K_2_CO_3_, DMSO, 60 °C, 3 h.

### 2.2. Cytotoxicity

The *in vitro* antitumor activity was evaluated on the K562, HepG2 and HT-29 cancer cell lines by the MTT assay, using HCPT as the reference compound. As shown in Table 1, the conjugates containing valine spacers (compounds **4a**–**4f**) exhibited comparable or more potent (2–5 times) antiproliferative activity than HCPT. The most potent monovalent compound was **4a**, which contained a short mPEG chain, while **4b** and **4c** possessed comparable activity to HCPT. The IC_50_ values of **4d**–**4f** were almost the same as that of **4a**. Compared to the valine-linked conjugates, the activity of compounds **5** and **6** were significantly reduced, especially for **6**.

### 2.3. Stability and Conversion

The stability of these conjugates was studied by HPLC at pH 7.4 in Phosphate Buffered Saline (PBS) at 37 °C (Table 2). The results are showed in Table 2, the conjugates’ stability is closely related to the length of PEG and the liker type. For compounds **4a**–**4f** with valine spacers, the short PEG linked conjugates (**4a** and **4f**) exhibited controlled release of HCPT. The monovalent conjugate **4a** released HCPT at a slower rate than **4b**–**4e**. Surprisingly, the bivalent conjugate **4f** released HCPT very slowly, even slower than compound **6** which possessed an ether bond. The difference in stability of conjugates with different PEG chain lengths via carbamates (compounds **4a**–**4f**) maybe resulted from their different conformations in solution.

**Table 1:** Antiproliferative activity of the conjugates *in vitro.*

Compound	IC_50_ (µM)		
	K562	HepG2	HT-29
HCPT	0.11 ± 0.07	0.08 ± 0.02	0.22 ± 0.09
**4a**	0.02 ± 0.01	0.04 ± 0.01	0.04 ± 0.01 *
**4b**	0.12 ± 0.03	0.11 ± 0.06	0.19 ± 0.10
**4c**	0.06 ± 0.03	0.12 ± 0.08	0.21 ± 0.10
**4d**	0.02 ± 0.01	0.04 ± 0.02	0.04 ± 0.01 *
**4e**	0.03 ± 0.01	0.05 ± 0.03 *	0.14 ± 0.09
**4f**	0.02 ± 0.01	0.05 ± 0.01	0.04 ± 0.01 *
**5**	0.24 ± 0.05 *	0.39 ± 0.23	0.42 ± 0.06 *
**6**	26.99 ± 0.74 **	32.30 ± 1.48 **	42.97 ± 16.67 **

* *p* < 0.05; ** *p* < 0.01, compared with the control group (HCPT).

**Table 2:** Stability of the conjugates at pH 7.4.

Compound	Released HCPT (%)			
	1 h	2 h	3 h	4 h
**4a**	75	80	82	82
**4b**	96	100	-	-
**4c**	100	-	-	-
**4d**	70	90	94	96
**4e**	100	-	-	-
**4f**	20	32	35	38
**5**	54	65	68	68
**6**	33	45	54	56

### 2.4. Discussion

In this study, a series of novel conjugates of HCPT were designed and synthesized using PEG as carrier. The water solubility of all the compounds was significantly improved by introducing the water-soluble PEG chain. Considering the stability and the antitumor activity together, it was supposed that the active form of monovalent compounds **4b** and **4c** was HCPT, since they released almost 100% of their HCPT in a very short time and exhibited comparable IC_50_ values as HCPT. Similarly, bivalent **4d** and **4e** also released HCPT very fast, but their IC_50_ values are lower than HCPT, which is probably mainly due to the bivalence effect. Compounds **5** and **6**, showed reduced activity (especially **6**), maybe because their HCPT release rates are significantly reduced and they didn’t exhibited cytotoxity by themselves, while conjugates **4a** and **4f**, linked with relatively short PEGs, showed excellent activity and controlled release of HCPT. The reason that conjugates **4a** and **4f** released HCPT more slowly is probably because their conformations differ from other conjugates containing longer PEG chains and valine spacers (compounds **4b**–**4e**), while they exhibited better activity than HCPT perhaps because **4a** and **4f** could directly interact with topoisomerase I by themselves too. Of course, this speculation needs to be further confirmed.

## 3. Experimental Section

### 3.1. General Information

^1^H-NMR spectra were recorded on a Bruker AM-400 NMR spectrometer (Zurich, Switzerland) in DMSO-*d_6_*. The chemical shifts are reported in δ (ppm) relative to tetramethylsilane as the internal standard. The mass spectra were obtained on a Bruker MALDI-TOF mass spectrometer (Billerica, MA, USA). Thin-layer chromatography (TLC) was performed using E. Merck silica gel 60 GF_254_ precoated plates (Darmstadt, Germany) and visualized using a combination of UV254 and UV365 lamps. Silica gel (particle size 200–400 mesh, Marine Chemical Group Co., Qingdao, China) was used for flash chromatography.

### 3.2. Synthesis

*4-Nitrophenyl camptothecin-10-yl carbonate* (**1a**): To a stirred solution of 10-hydroxycamptothecin (3.0 g, 8.2 mmol) in dry THF (600 mL) at 0 °C was added triethylamine (11.5 mL, 82.3 mmol) followed by 4-nitrophenyl chloroformate (6.6 g, 32.9 mmol). The reaction mixture was stirred at room temperature for 1 h after which the solvents were then removed under vacuum to give a brownish residue that was purified by column chromatography (200 mesh silica gel, EtOAc) to afford a crude product. Compound **1a** (3.59 g, 82% yield) was purified by recrystallization with hexane and dichloromethane. ^1^H-NMR (DMSO-*d_6_*) δ (ppm): 8.74 (s, 1H), 8.40 (d, *J* = 9.2 Hz, 2H), 8.30 (d, *J* = 9.2 Hz, 1H), 8.20 (d, *J* = 2.4 Hz, 1H), 7.97–7.80 (m, 1H), 7.78 (d, 2H, *J* = 9.2 Hz), 7.37 (s, 1H), 6.54 (s, 1H), 5.44 (s, 2H), 5.32 (s, 2H), 1.85–1.92 (m, 2H), 0.89–0.92 (m, 3H).

*MPEG-Val(Boc)* (**2a**–**2c**) *and Val(Boc)-PEG-Val(Boc)* (**2d**–**2f**): *N*-Boc-l-valine (1.2 g, 5.5 mmol) was dissolved in dry CH_2_Cl_2_ (15 mL). Next 4-dimethylaminopyridine (DMAP, 76.0 mg, 0.6 mmol) and 1-ethyl-3-(3-dimethylaminopropyl)carbodiimide hydrochloride (1.2 g, 6.0 mmol) were added at 0 °C, and the mixture was stirred for 10 min after which, mPEG or PEG (2.5 mmol) was added, and the resulting mixture was stirred overnight at room temperature. After the reaction was complete, the mixture was washed with 0.1 N HCl and brine, and dried with anhydrous sodium sulfate. Removal of solvent under vacuum gave a residue that was purified by recrystallization from ethyl ether to afford compounds **2a**–**2f** (85%–95%).

*MPEG-Val* (**3a**–**3c**) *and Val-PEG-Val* (**3d**–**3f**): To stirred solutions of **2a**–**2f** in CH_2_Cl_2_ (5 mL) was added trifluoroacetic acid (TFA, 4.6 mL) dropwise. The mixture were stirred at room temperature for 1 h, eluted with water, pH adjusted to 8 by adding aq. NaHCO_3_, and extracted with CH_2_Cl_2_. The organic layers were combined and dried with anhydrous sodium sulfate. Removal of solvent under vacuum and recrystallization with ethyl ether gave compounds **3a**–**3f** (87%–94% yield).

*MPEG_350_-Val* (**3a**): ^1^H-NMR (DMSO-*d_6_*) δ (ppm): 4.18–4.23 (m, 1H), 4.09–4.15 (m, 1H), 3.62–3.41 (s, mPEG), 3.24 (s, 3H), 3.14 (d, *J* = 5.6 Hz, 1H), 1.96 (s, 2H), 1.88–1.82 (m, 1H), (d, *J* = 6.8 Hz, 3H), 0.83 (d, *J* = 6.8 Hz, 3H).

*MPEG_2000_-Val* (**3b**): ^1^H-NMR (DMSO-*d_6_*) δ (ppm): 4.18–4.23 (m, 1H), 4.09–4.15(m, 1H), 3.69–3.41 (s, mPEG), 3.24 (s, 3H), 3.15 (d, *J* = 8.0 Hz, 1H), 2.05 (s, 2H), 1.87–1.82 (m, 1H), 0.88 (d, *J* = 6.8 Hz, 3H), 0.83 (d, *J* = 6.8 Hz, 3H).

*MPEG_5000_-Val* (**3c**): ^1^H-NMR (DMSO-*d_6_*) δ (ppm): 4.18–4.23 (m, 1H), 4.09–4.15 (m, 1H), 3.78–3.36 (s, PEG), 3.24 (s, 3H, CH_3_O-), 3.15 (d, *J* = 5.3 Hz, 1H), 1.86–1.80 (m, 1H), 0.88 (d, *J* = 6.8 Hz, 3H), 0.83 (d, *J* = 6.8 Hz, 3H).

*Val-PEG_2000_-Val* (**3d**): ^1^H-NMR (DMSO-*d_6_*) δ (ppm): 4.18–4.23 (m, 2H), 4.09–4.14 (m, 2H), 3.69–3.41 (s, PEG), 3.13 (d, *J* = 5.2 Hz, 2H), 1.86–1.81 (m, 2H), 1.76 (s, 4H), 0.87 (d, *J* = 6.8 Hz, 6H), 0.82 (d, *J* = 6.8 Hz, 6H).

*Val-PEG_20000_-Val* (**3e**): ^1^H-NMR (DMSO-*d_6_*) δ (ppm): 4.12–4.21 (m, 4H), 3.70–3.35 (s, PEG), 3.15 (d, *J* = 4.0 Hz, 2H), 1.86–1.90 (m, 6H), 0.90 (d, *J* = 6.6 Hz, 6H), 0.85 (d, *J* = 6.6 Hz, 6H).

*Val-PEG_300_-Val* (**3f**): ^1^H-NMR (DMSO-*d_6_*) δ (ppm): 4.10–4.23 (m, 4H), 3.60 (t, *J* = 4.8 Hz, 2H), 3.51 (s, PEG), 3.12 (d, *J* = 5.6 Hz, 2H), 1.79–1.86 (m, 6H), 0.87–0.89 (dd, *J* = 6.4, 4.0 Hz, 6H), 0.82–0.84 (d, *J* = 6.8 Hz, 6H).

*MPEG-Val-HCPT* (**4a**–**4c**) *and HCPT-Val-PEG-Val-HCPT* (**4d**–**4f**): Compound **1a** (0.6 g, 1.1 mmol) was dissolved in anhydrous DMF (3 mL) and CH_2_Cl_2_ (3 mL). Next, **3a**–**3f** (0.7 mmol) were added to make individual mixtures that were then stirred at room temperature. After reacting for 1 h, the solutions were washed with brine and the organic layers were combined and dried with anhydrous sodium sulfate. Removal of solvent under vacuum and column chromatography (200 mesh silica gel) with CH_2_Cl_2_/CH_3_OH (30:1, v/v) as eluent gave the crude products. Compounds **4a**–**4f** (63%–67% yield) were obtained by recrystallization from ethyl ether.

*MPEG_350_-Val-HCPT* (**4a**): ^1^H-NMR (DMSO-*d_6_*) δ (ppm): 8.68 (s, 1H), 8.43 (d, *J* = 8.0 Hz, 1H), 8.18 (d, *J* = 9.2 Hz, 1H), 7.92 (d, *J* = 2.0 Hz, 1H), 7.63–7.66 (dd, *J* = 9.2, 2.4 Hz, 1H), 7.34 (s, 1H), 6.52 (s, 1H), 5.43 (s, 2H), 5.30 (s, 2H), 4.28–4.33 (m, 1H), 4.18–4.22 (m, 1H), 4.03–4.07 (m, 1H), 3.49–3.66 (s, mPEG), 3.23 (s, 3H), 2.14–2.17 (m, 1H), 1.84 –1.91 (m, 2H), 0.98–1.01 (m, 6H), 0.89 (t, *J* = 7.2Hz, 3H).

*MPEG_2000_-Val-HCPT* (**4b**): ^1^H-NMR (DMSO-*d_6_*) δ (ppm): 8.68 (s, 1H), 8.44 (d, *J* = 8.4 Hz, 1H), 8.19 (d, *J* = 9.2 Hz, 1H), 7.92 (d, *J* = 2.4 Hz, 1H), 7.63–7.66 (dd, *J* = 9.2, 2.8 Hz, 1H), 7.35 (s, 1H), 6.53 (s, 1H), 5.43 (s, 2H), 5.30 (s, 2H), 4.28–4.32 (m, 1H), 4.17–4.21 (m, 1H), 4.02–4.06 (m, 1H), 3.41–3.68 (s, mPEG), 3.24 (s, 3H), 2.13–2.18 (m, 1H), 1.84–1.91 (m, 2H), 0.98–1.01 (m, 6H), 0.89 (t, *J* = 7.2 Hz, 3H).

*MPEG_5000_-Val-HCPT* (**4c**): ^1^H-NMR (DMSO-*d_6_*) δ (ppm): 8.69 (s, 1H), 8.44 (d, *J* = 8.0 Hz, 1H), 8.19 (d, *J* = 8.8 Hz, 1H), 7.91 (s, ,1H), 7.65 (d, *J* = 7.6 Hz, 1H), 7.35 (s, 1H), 6.54 (s, 1H), 5.43 (s, 2H), 5.30 (s, 2H), 4.29–4.32 (m, 1H), 4.17–4.20 (m, 1H), 4.03–4.06 (m, 1H), 3.44–3.68 (s, mPEG), 3.24 (s, 3H), 2.14–2.18 (m, 1H), 1.86–1.89 (m, 2H), 0.98–1.01 (m, 6H), 0.89 (t, *J* =7.2 Hz, 3H).

*HCPT-Val-PEG_2000_-Val-HCPT* (**4d**): ^1^H-NMR (DMSO-*d_6_*) δ (ppm): 8.68 (s, 2H), 8.43 (d, *J* = 8.0 Hz, 2H), 8.19 (d, *J* = 9.2 Hz, 2H), 7.91 (d, *J* = 2.4 Hz, 2H), 7.63–7.66 (dd, *J* = 9.2, 2.4 Hz, 2H), 7.35 (s, 2H), 6.53 (s, 2H), 5.43 (s, 4H), 5.30 (s, 4H), 4.28–4.32 (m, 2H), 4.17–4.20 (m, 2H), 4.02–4.06 (m, 2H), 3.45–3.66 (s, PEG), 2.13–2.18 (m, 2H), 1.84–1.91 (m, 4H), 0.98–1.01 (m,12H), 0.89 (t, *J* = 7.2 Hz, 6H).

*HCPT-Val-PEG_20000_-Val-HCPT* (**4e**): ^1^H-NMR (DMSO-*d_6_*) δ (ppm): 8.70 (s, 2H), 8.45 (d, *J* = 7.2 Hz, 2H), 8.20 (d, *J* = 8.8 Hz, 2H), 7.93 (s, 2H), 7.65–7.67 (d, *J* = 7.6, 2H), 7.37 (s, 2H), 6.55 (s, 2H), 5.44 (s, 4H), 5.32 (s, 4H), 4.21–4.31 (m, 4H), 4.06–4.08 (m, 2H), 3.52–3.69 (s, PEG), 2.17–2.18 (m, 2H), 1.89 (m, 4H), 1.01 (m, 12H), 0.83 (t, *J* = 7.2Hz, 6H).

*HCPT-Val-PEG300-Val-HCPT* (**4f**): ^1^H-NMR (DMSO-*d_6_*) δ (ppm): 8.66 (s, 2H), 8.44 (d, *J* = 8.0 Hz, 2H), 8.17 (d, *J* = 8.8 Hz, 2H), 7.90 (s, 2H), 7.63 (d, *J* = 8.4, 2H), 7.34 (s, 2H), 6.53 (s, 2H), 5.43 (s, 4H), 5.28 (s, 4H), 4.18–4.33 (m, 4H), 4.03–4.07 (m, 2H), 3.50–3.65 (s, PEG), 2.16–2.17 (m, 2H), 1.85–1.91 (m, 4H), 0.98–1.00 (m, 12H), 0.89 (t, *J* = 7.2 Hz, 6H).

*MPEG_2000_ (4-nitrophenyl) carbonate* (**1b**): Methoxypolyethylene glycol 2000 (mPEG_2000_, 3.0 g, 1.5 mmol) was dissolved in anhydrous CH_2_Cl_2_ (15 mL). Triethylamine (4.2 mL, 30.0 mmol) and 4-nitrophenyl chloroformate (1.2 g, 6.0 mmol) were added at 0 °C and the mixture was stirred at room temperature for 3 h. The mixture was concentrated under vacuum, purified by column chromatography (CH_2_Cl_2_/CH_3_OH, 25:1, v/v) and recrystallized from ethyl ether to afford compound **1b** (2.6 g, 80% yield). ^1^H-NMR (DMSO-*d_6_*) δ (ppm): 8.32(d, *J* = 9.2 Hz, 2H), 7.57(d, *J* = 9.2 Hz, 2H), 4.38–4.36 (m, 2H), 3.71–3.42 (s, mPEG), 3.24 (s, 3H).

*mPEG_2000_-OCO-HCPT* (**5**): To a stirred solution of HCPT (150.0 mg, 0.4 mmol) in anhydrous DMF were added triethylamine (0.24 mL, 1.7 mmol) and compound **1b** (0.6 g, 0.3 mmol). The reaction mixture was stirred at 60 °C for 45 min, diluted with water, and extracted with CH_2_Cl_2_. The organic layer was combined, dried with anhydrous sodium sulfate, concentrated under vacuum, purified by column chromatography (CH_2_Cl_2_/CH_3_OH, 25:1, v/v) and recrystallized with ethyl ether to give compound **5** (350.0 g, 55% yield). ^1^H-NMR (DMSO-*d_6_*) δ (ppm): 8.70 (s, 1H), 8.24 (d, *J* = 9.2 Hz, 1H), 8.05 (d, *J* = 2.4 Hz, 1H), 7.78–7.81 (dd, *J* = 9.2, 2.4 Hz, 2H), 7.36 (s, 1H), 6.55 (s, 1H), 5.44 (s, 2H), 5.31 (s, 2H), 4.39 (t, 2H), 3.75–3.41 (s, mPEG), 3.24 (s, 3H), 1.82–1.93 (m, 2H), 0.89 (t, *J* = 7.2 Hz, 3H).

*MPEG_2000_ 4-methylbenzenesulfonate* (**7**): mPEG_2000_ (3.0 g, 1.5 mmol) was dissolved in anhydrous CH_2_Cl_2_ (15.0 mL). Triethylamine (4.2 mL, 29.8 mmol) and 4-methoxybenzenesulfonyl chloride (0.9 g, 5.1 mmol) were added at 0 °C. The mixture was heated to reflux for 5 h, diluted with CH_2_Cl_2_, and washed with 1 N HCl and brine. The organic layer was dried with anhydrous sodium sulfate, concentrated under vacuum and purified by recrystallization with ethyl ether to give compound **7** (3.0 g, 91% yield). ^1^H-NMR (DMSO-*d_6_*) δ (ppm): 7.78 (d, *J* = 8.4 Hz, 2H), 7.48 (d, *J* = 8.0 Hz, 2H), 4.11 (t, *J* = 4.4 Hz, 2H), 3.69–3.45 (s, mPEG), 3.24 (s, 3H), 2.42 (s, 3H).

*1-Bromo mPEG_2000_* (**8**): To a solution of compound **7** (3.0 g, 1.4 mmol) in dry acetone (15.0 mL) was added LiBr (0.6 g, 7.0 mmol). The mixture was refluxed overnight, concentrated under vacuum, purified by column chromatography (200 mesh silica gel, CH_2_Cl_2_/CH_3_OH, 25:1, v/v), and recrystallized with ethyl ether to give compound **8** (2.3 g, 80% yield). ^1^H-NMR (DMSO-*d_6_*) δ (ppm): 3.80–3.77 (t, *J* = 5.8 Hz, 2H), 3.75–3.73 (t, *J* = 4.8 Hz, 2H), 3.69–3.45 (s, mPEG), 3.30 (s, 3H, CH_3_O-).

*mPEG_2000_-O-HCPT* (**6**): To a solution of HCPT (350.0 mg, 1.0 mmol) and potassium carbonate (270.0 mg, 2.0 mmol) in dry DMSO (2.5 mL) was added compound **8** (1.0 g, 0.5 mmol). The mixture was allowed to react for 3 h at 60 °C and the reaction mixture was directly loaded onto a silica gel column. Purification of the reaction mixture by column chromatography on silica gel using 4% CH_3_OH in CH_2_Cl_2_ as an eluent gave compound **6** (650.0 mg, 57% yield).^1^H-NMR (DMSO-*d_6_*) δ (ppm): 8.52 (s, 1H), 8.06 (d, *J* = 9.2 Hz, 1H), 7.53 (s, 1H), 7.51(d, *J* = 2.8 Hz, 1H) 7.28 (s, 1H), 6.50 (s, 1H), 5.42 (s, 2H), 5.26 (s, 2H), 4.29 (t, *J* = 4.2 Hz, 2H), 3.85 (t, *J* = 4.2 Hz, 2H), 3.41–3.68 (s, mPEG),3.24 (s, 3H), 1.83–1.90 (m, 2H), 0.89 (t, *J* = 7.2 Hz, 3H).

### 3.3. Cytotoxicity Study

The cytotoxicity of the target compounds were evaluated by the MTT assay. Cells were seeded at a density of 5 × 10^4^ cells/mL in 96-well microplates (100 μL/well). After 2 h for K562 cells and 24 h for HepG2 and HT-29 cells, media containing tested compounds were added in triplicate. After a 48 h incubation, the media were replaced with PBS medium containing 0.5 mg/mL MTT and the cells were incubated for another 4 h. Next, the medium was removed and 100 μL DMSO was added to each well to dissolve the formazan. The absorbance at 570/630 nm was measured for K562 cells and the absorbance at 490/630 nm was measured for HepG2 and HT-29 cells using a Thermo (Waltham, MA, USA) microplate reader. The untreated controls were considered as having a cell viability value of 100%. The half-maximal inhibiting concentration (IC_50_) values were obtained by nonlinear regression using GraphPad Prism 5.0. IC_50_ was measured three times for each compound.

### 3.4. Stability Test

The stability of all the compounds in PBS was examined by HPLC using a C18 analytical column. The mobile phase was composed of water and acetonitrile at a ratio of 60:40. The amount of the compound and its metabolite HCPT were flowed at a rate of 1.0 mL·min^−1^ and detected by a UV wavelength of 360 nm. Stability test in PBS: each compound was dissolved in PBS (pH 7.4), incubated at 37 °C and analyzed at 1, 2, 3, and 4 h.

### 3.5. Statistics

Values are IC_50_ (μM) means ± SEM of three separate experiments. All statistical analysis was performed by using GraphPad Prism software version 5.0 (GraphPad Software, Inc., San Diego, CA, USA). * *p* < 0.05; ** *p* < 0.01, compared with the control group (HCPT).

## 4. Conclusions

In order to improve the pharmacokinetic properties of HCPT and find new water soluble camptothecin type drugs with higher potency and lower side effects, eight novel conjugates of HCPT were designed and synthesized using different lengths of PEG as carriers and three types of linkages. The rate of release of HCPT and antitumor activity of the obtained conjugates are related to the length of the PEG chain and the linker type. Our results showed that conjugates with relatively short PEG chains and valine-spacers exhibited excellent antitumor activity and controlled release of HCPT. Further exploration of the mechanism on the influence of PEG length and different spacers on the HCPT release properties and antitumor activity is ongoing in our lab.

## Supplementary Materials

Supplementary materials can be accessed at: http://www.mdpi.com/1420-3049/20/05/9393/s1.
