# Supplementary material for: Synthesis and Biological Evaluation of Novel Water-Soluble Poly-(ethylene glycol)-10-hydroxycamptothecin Conjugates
Source: Molecules. 2015 May 21;20(5):9393–404. doi: 10.3390/molecules20059393 (PMC6272474; doi:10.3390/molecules20059393)
Supplement: Supplementary file 1 [file molecules-20-09393-s001.pdf]

Supplementary Materials

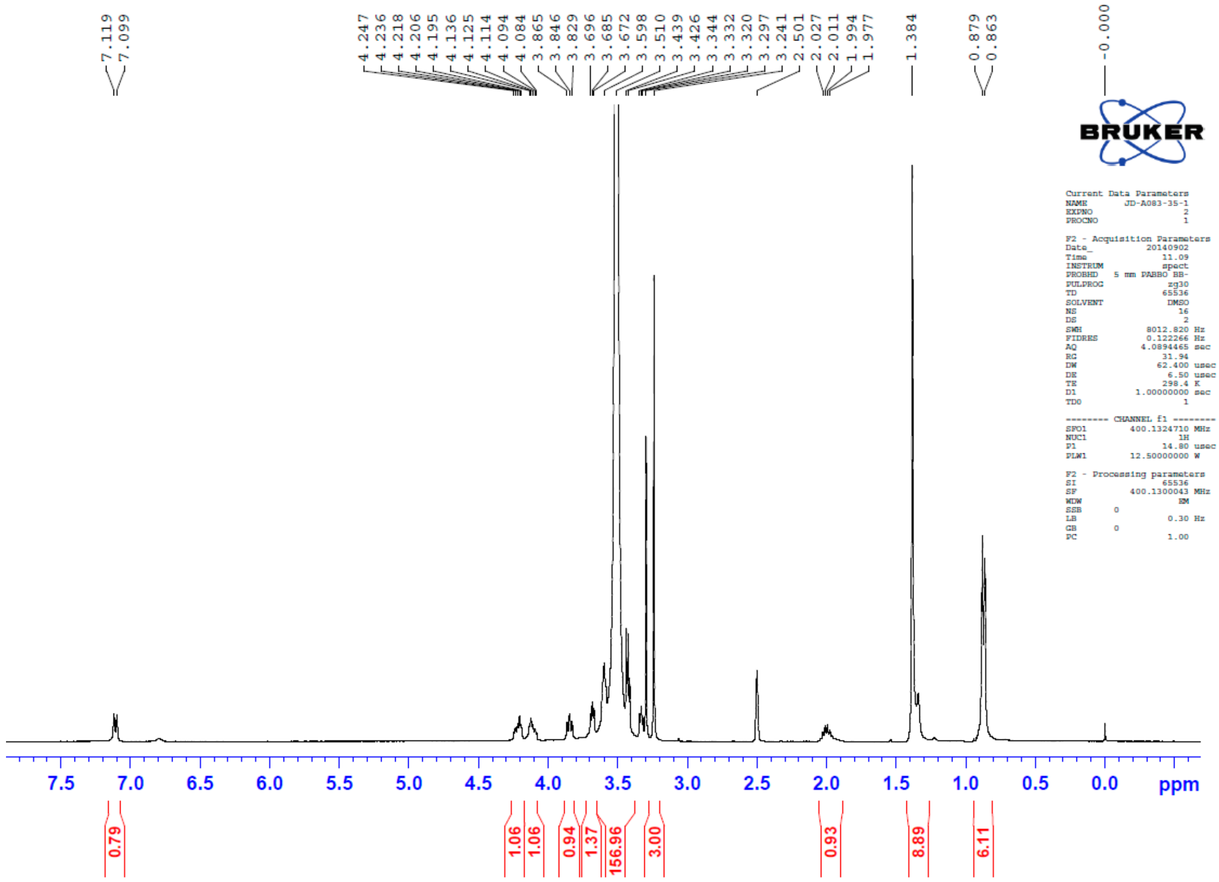

Figure S1. ^{1}H-NMR of compound **1a**.

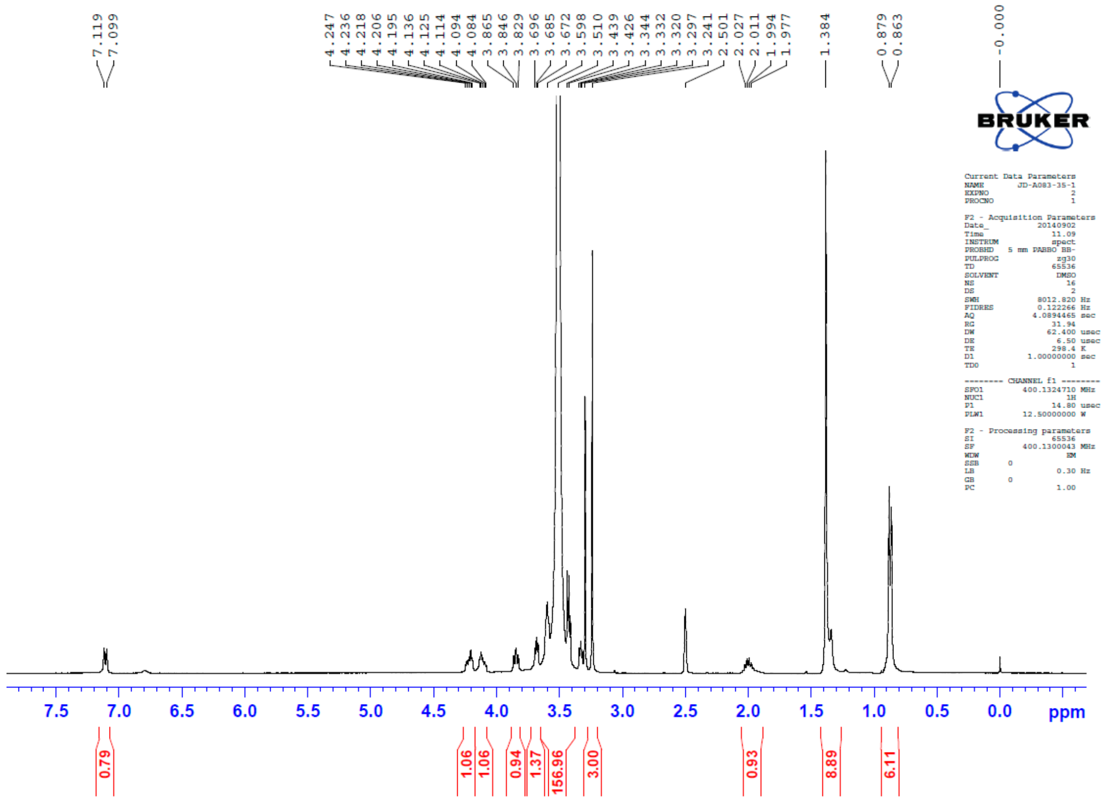

Figure S2. ^{1}H-NMR of compound **2b**.

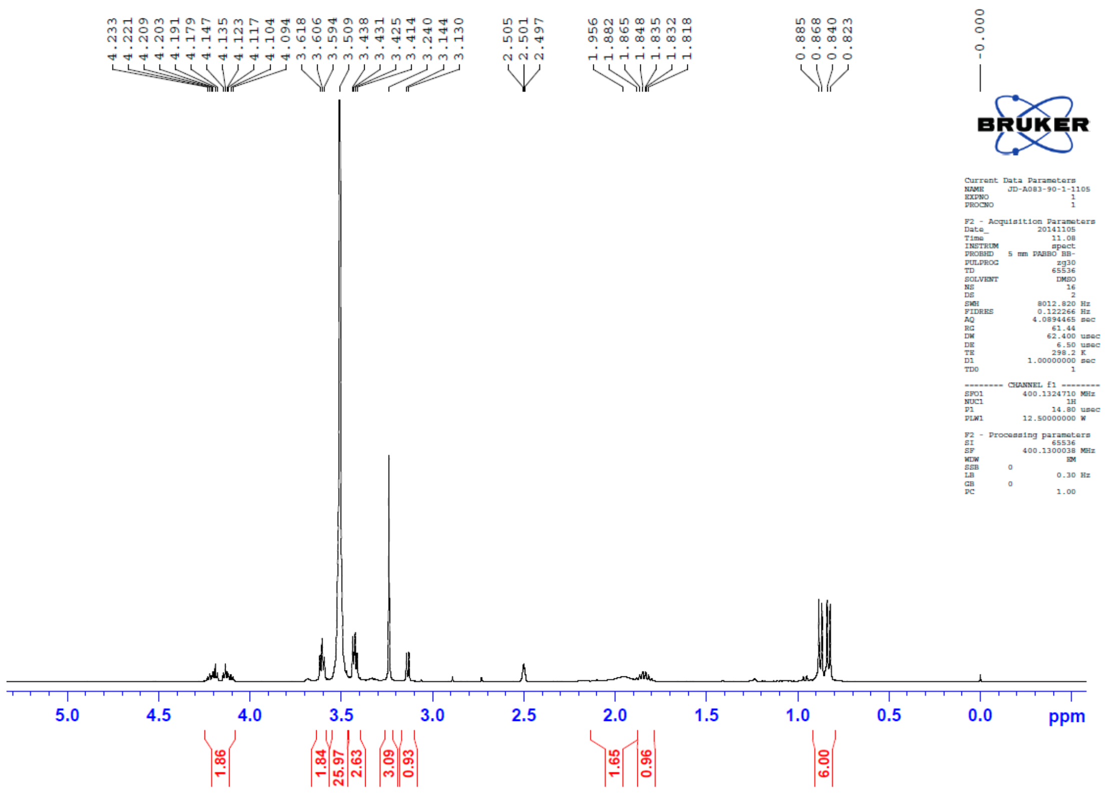

Figure S3. ^{1}H-NMR of compound **3a**.

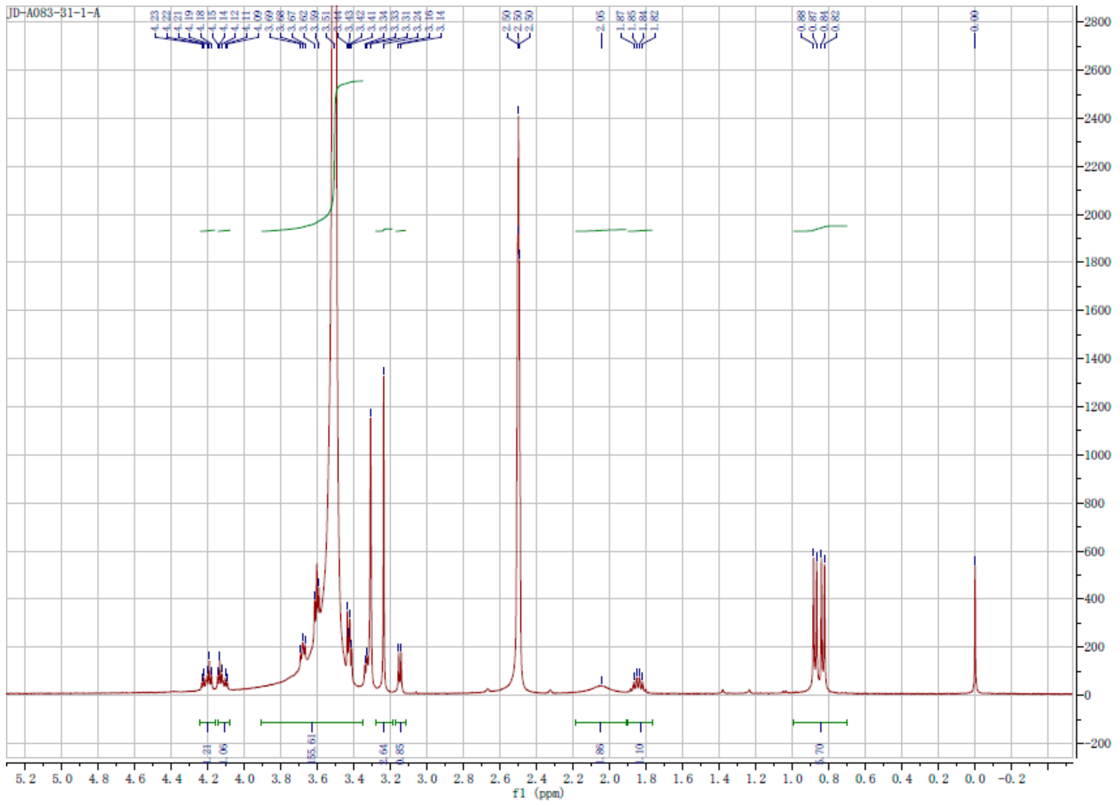

Figure S4. ^{1}H-NMR of compound **3b**.

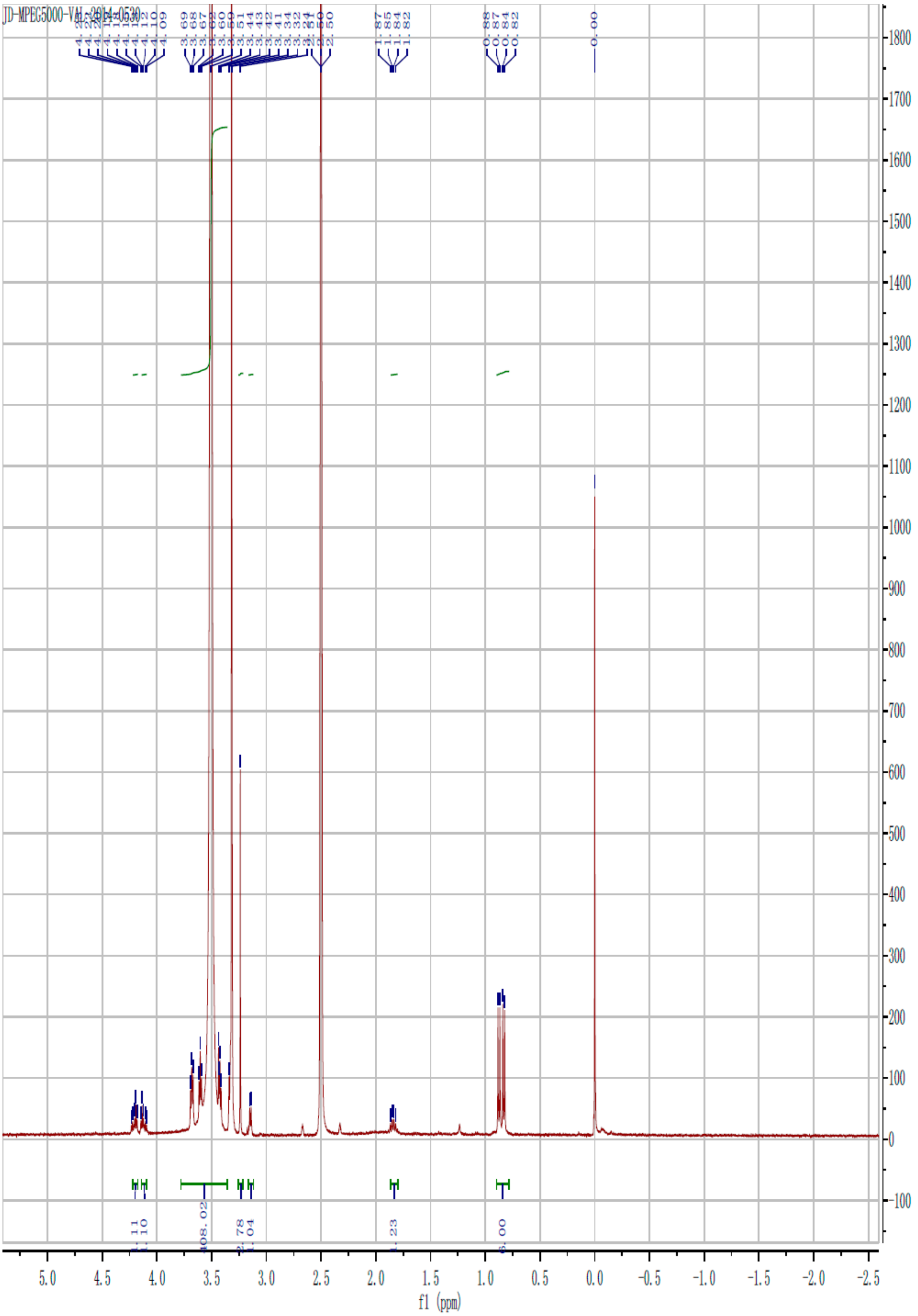

Figure S5. ^{1}H-NMR of compound **3c**.

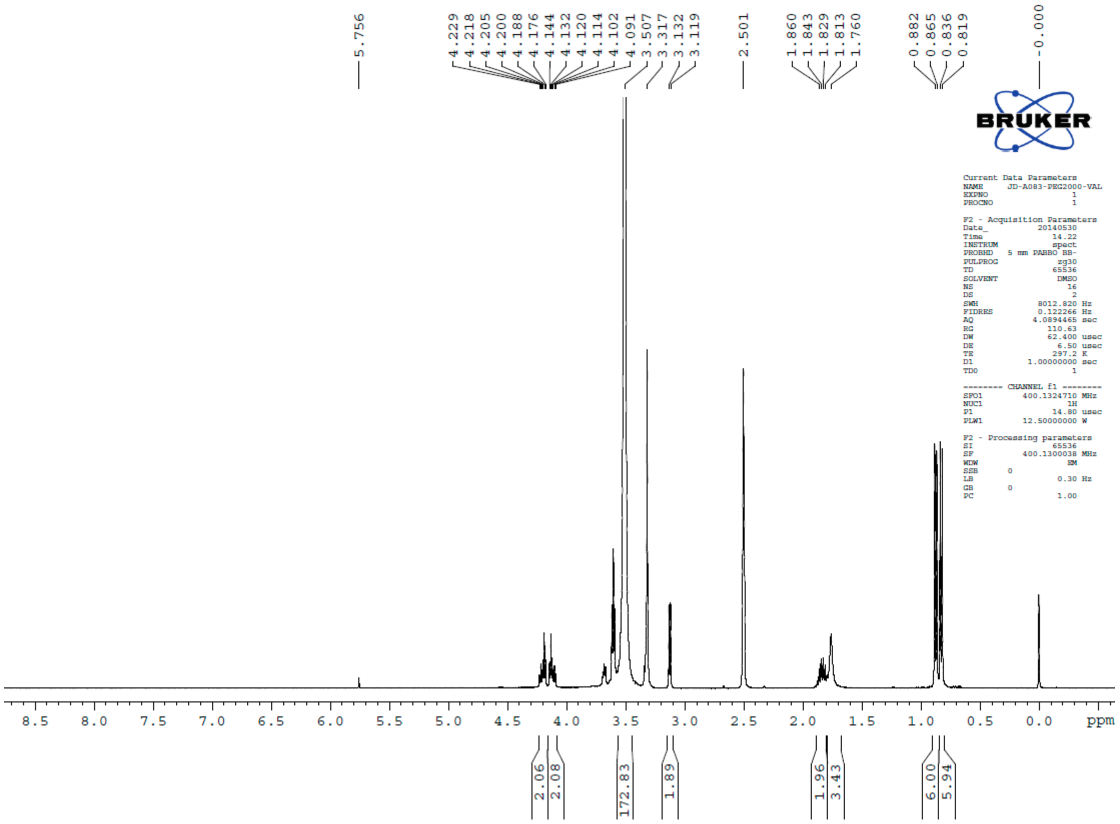

Figure S6. ^{1}H-NMR of compound **3d**.

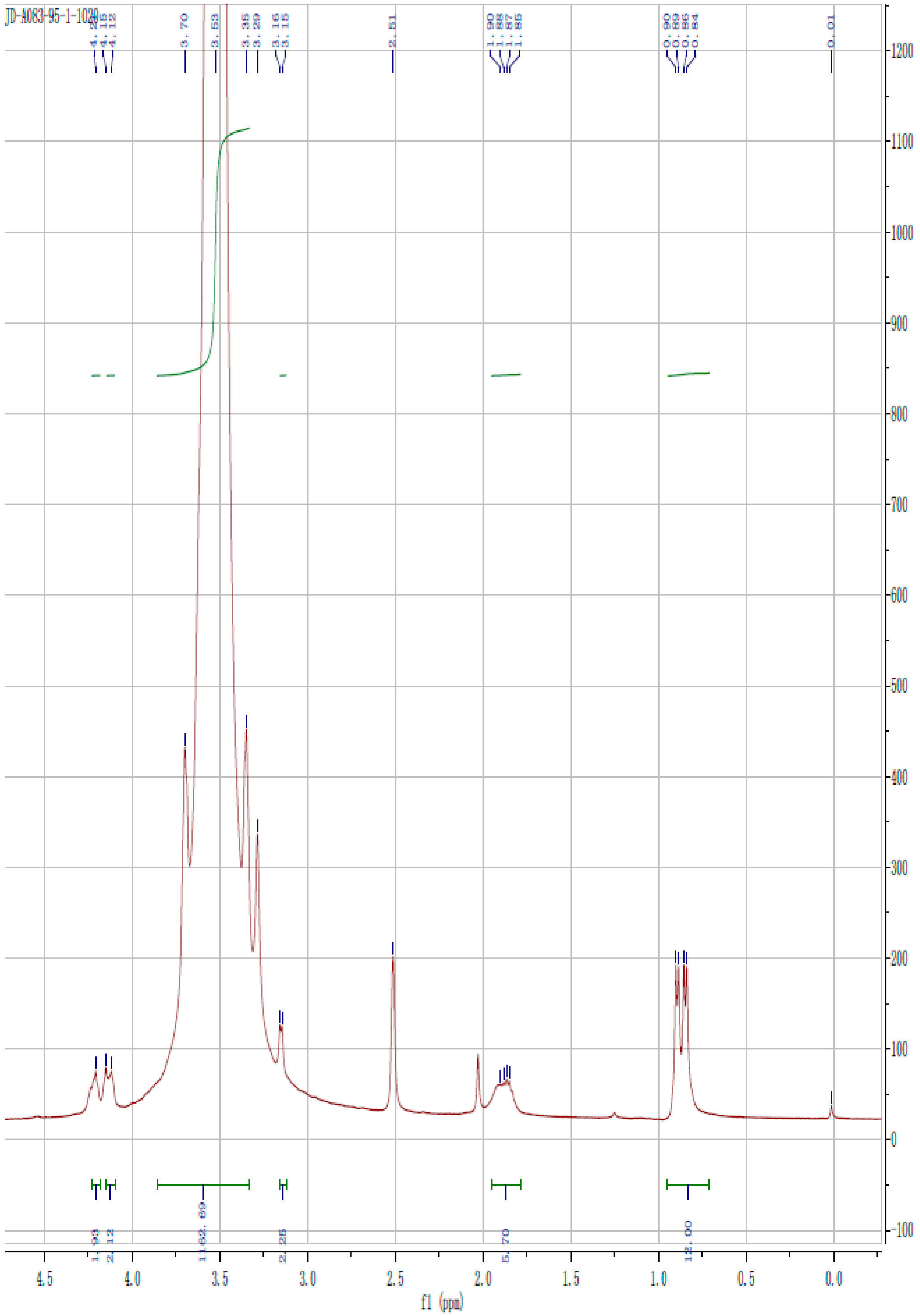

Figure S7. ^{1}H-NMR of compound **3e**.

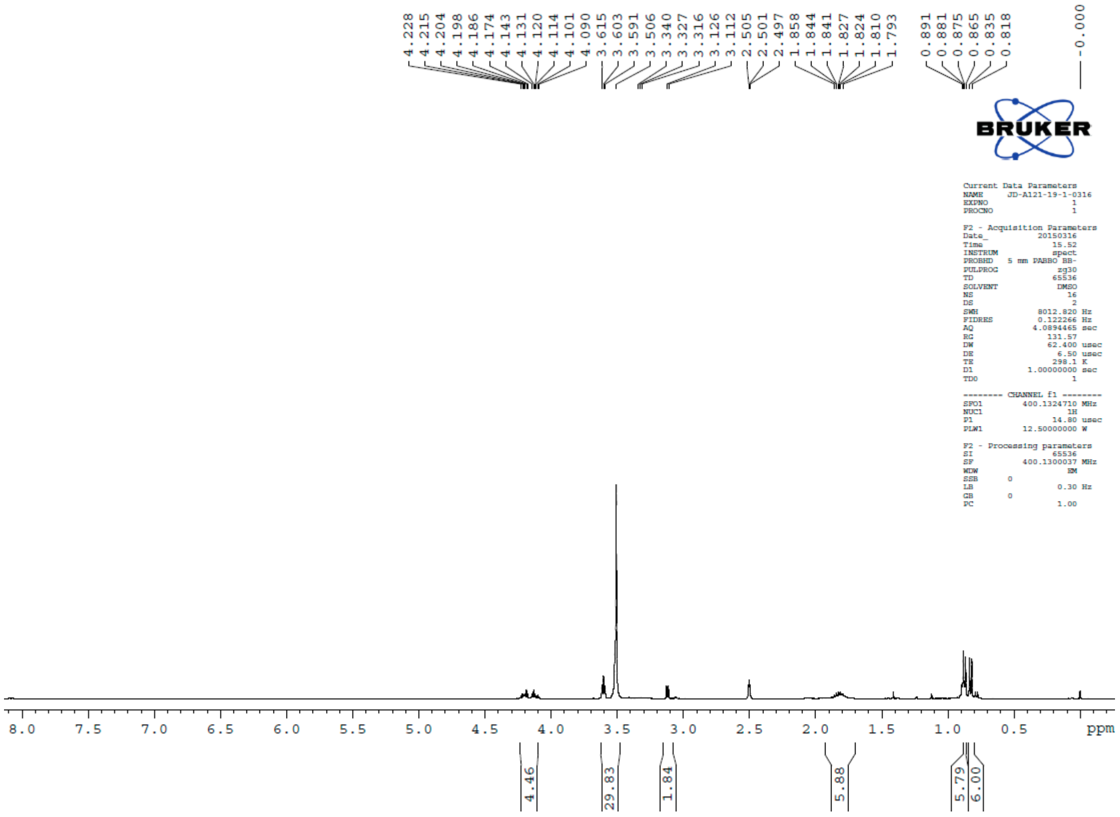

Figure S8. ^{1}H-NMR of compound **3f**.

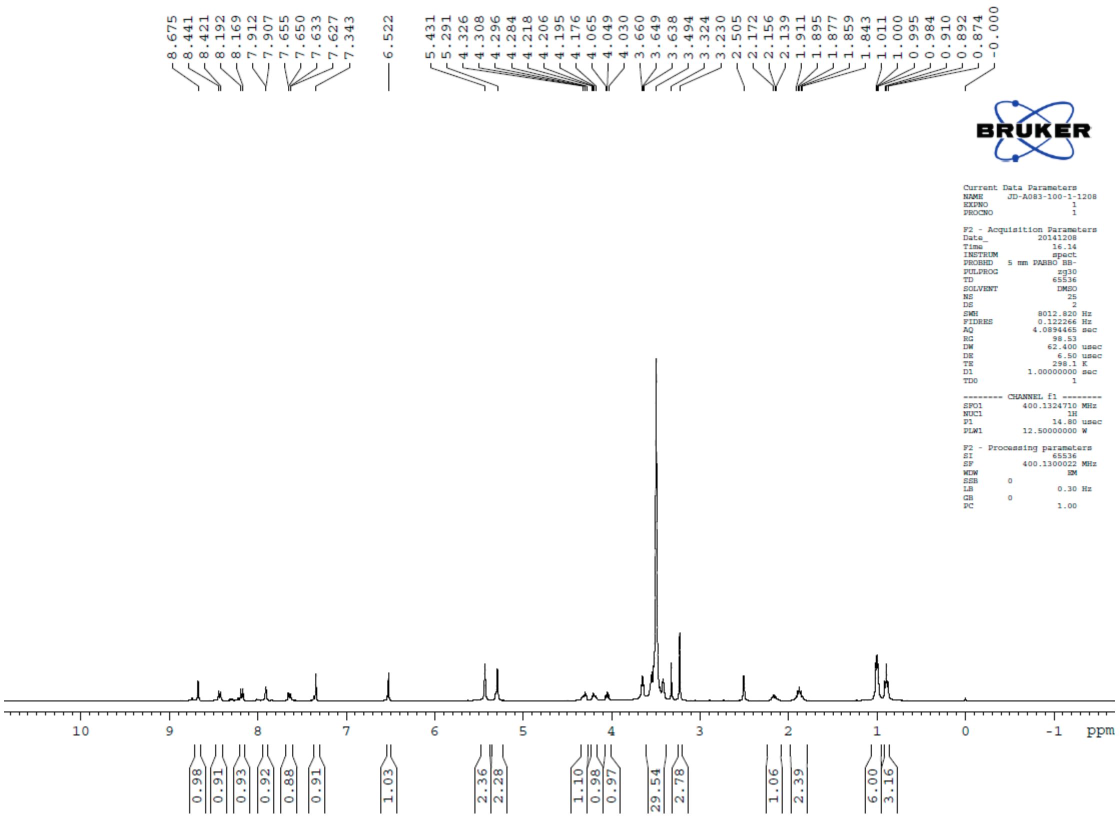

Figure S9. ^{1}H-NMR of compound **4a**.

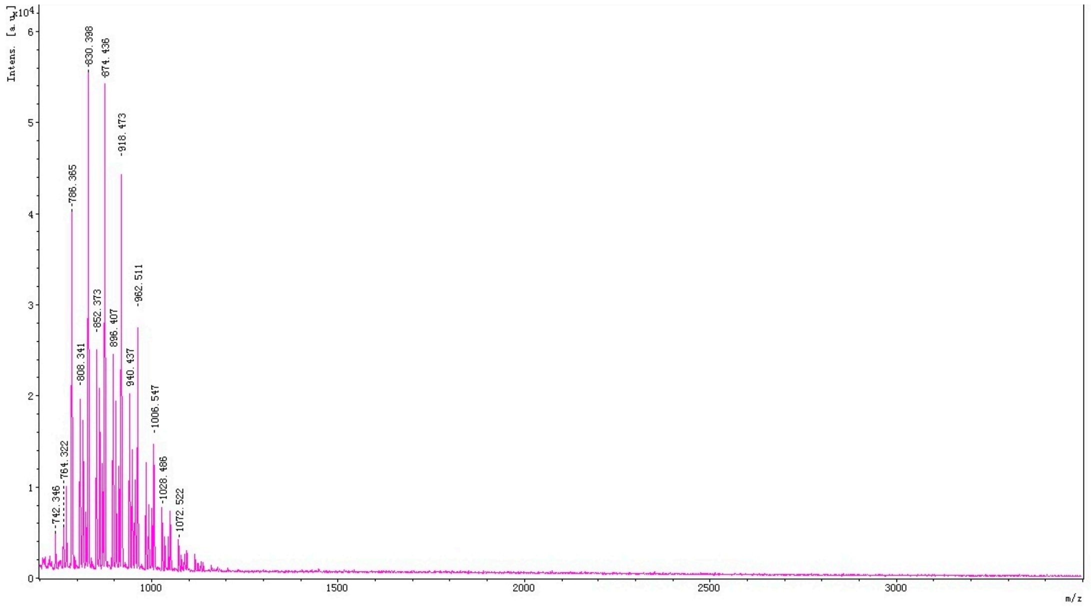

Figure S10. MALDI-TOF of compound **4a**.

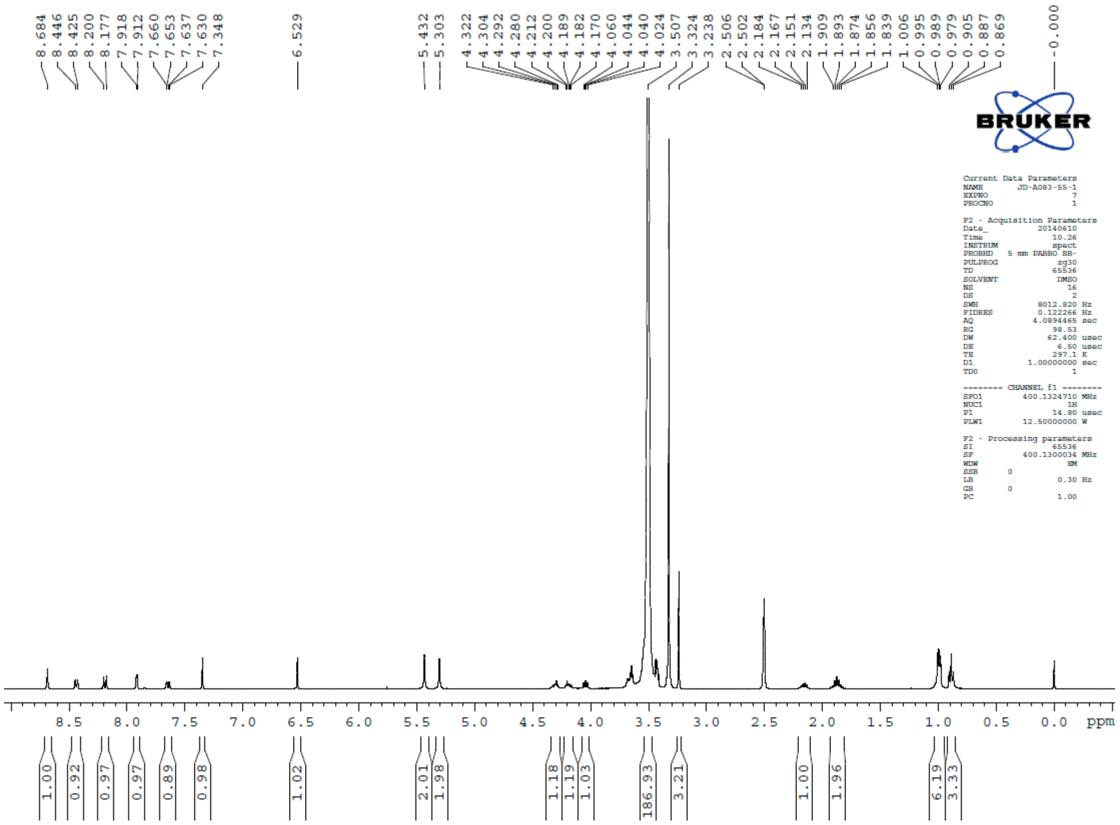

Figure S11. ^{1}H-NMR of compound **4b**.

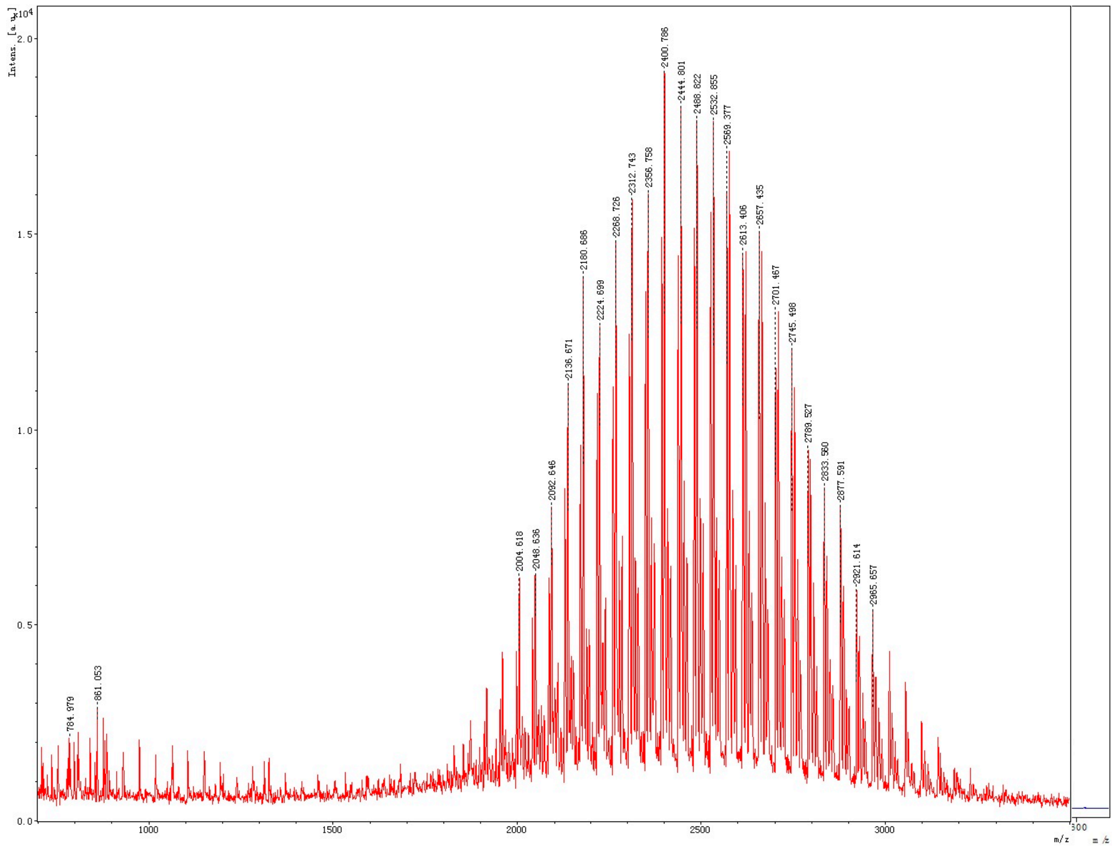

Figure S12. MALDI-TOF of compound **4b**.

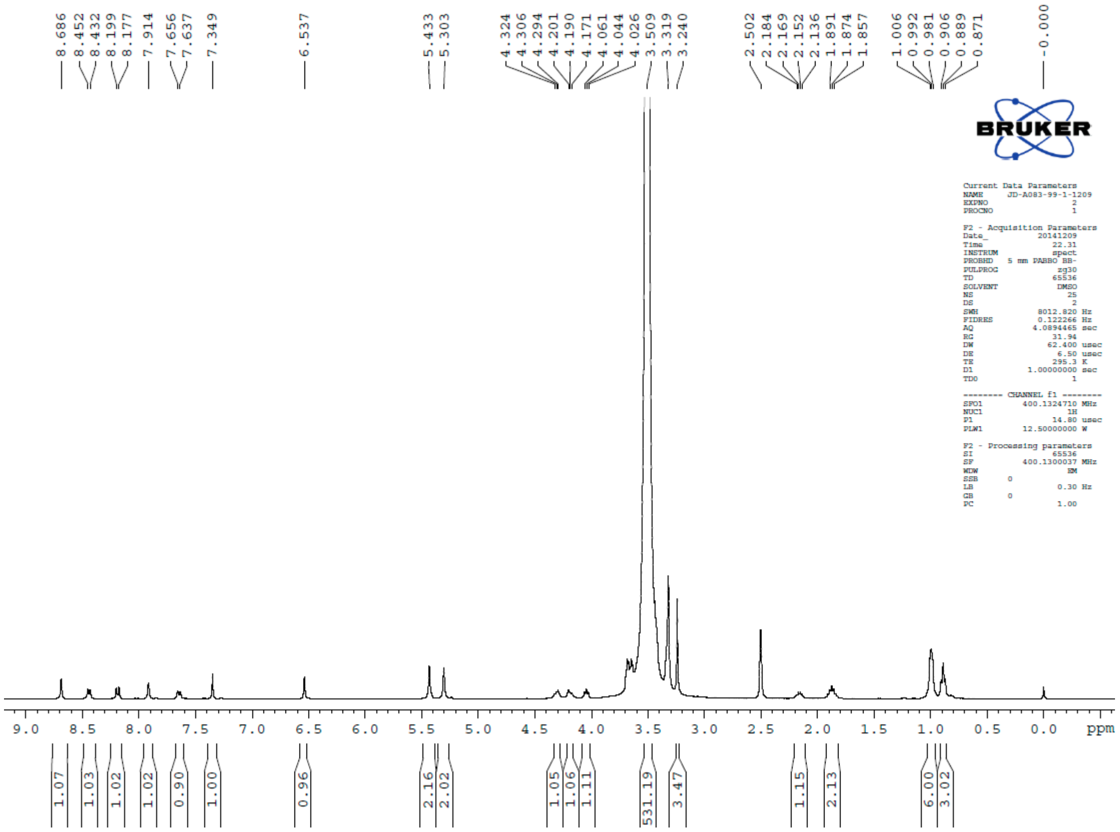

Figure S13. ^{1}H-NMR of compound **4c**.

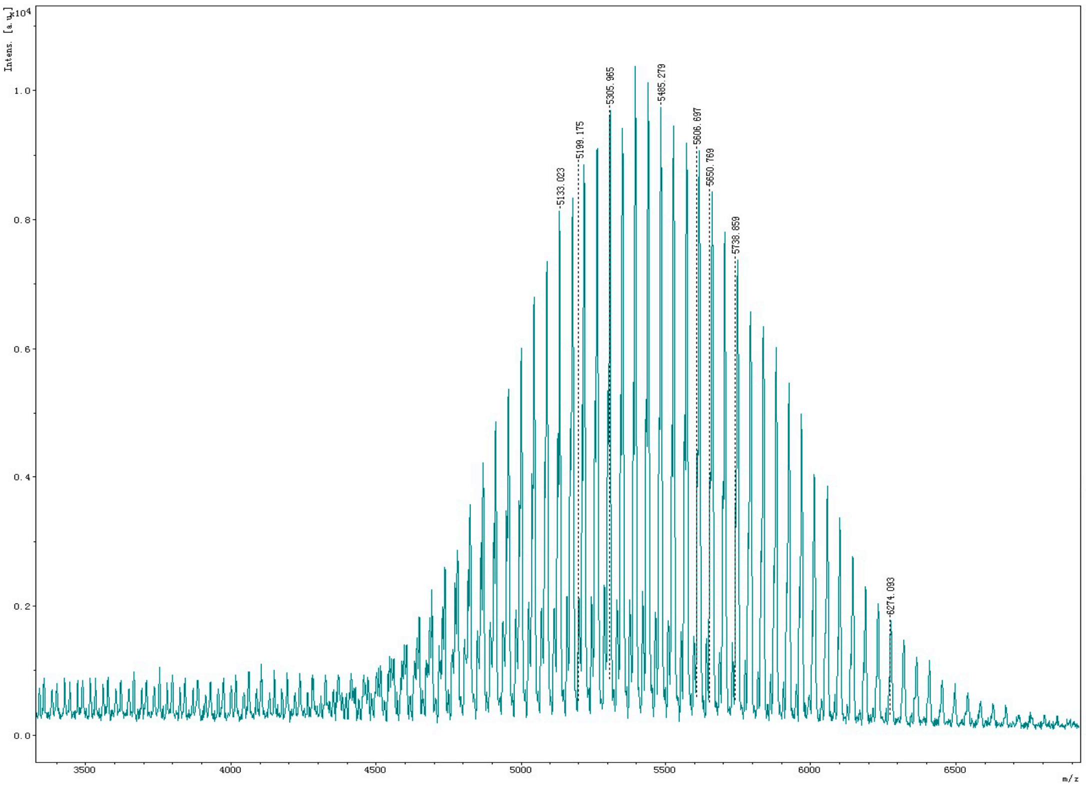

Figure S14. MALDI-TOF of compound **4c**.

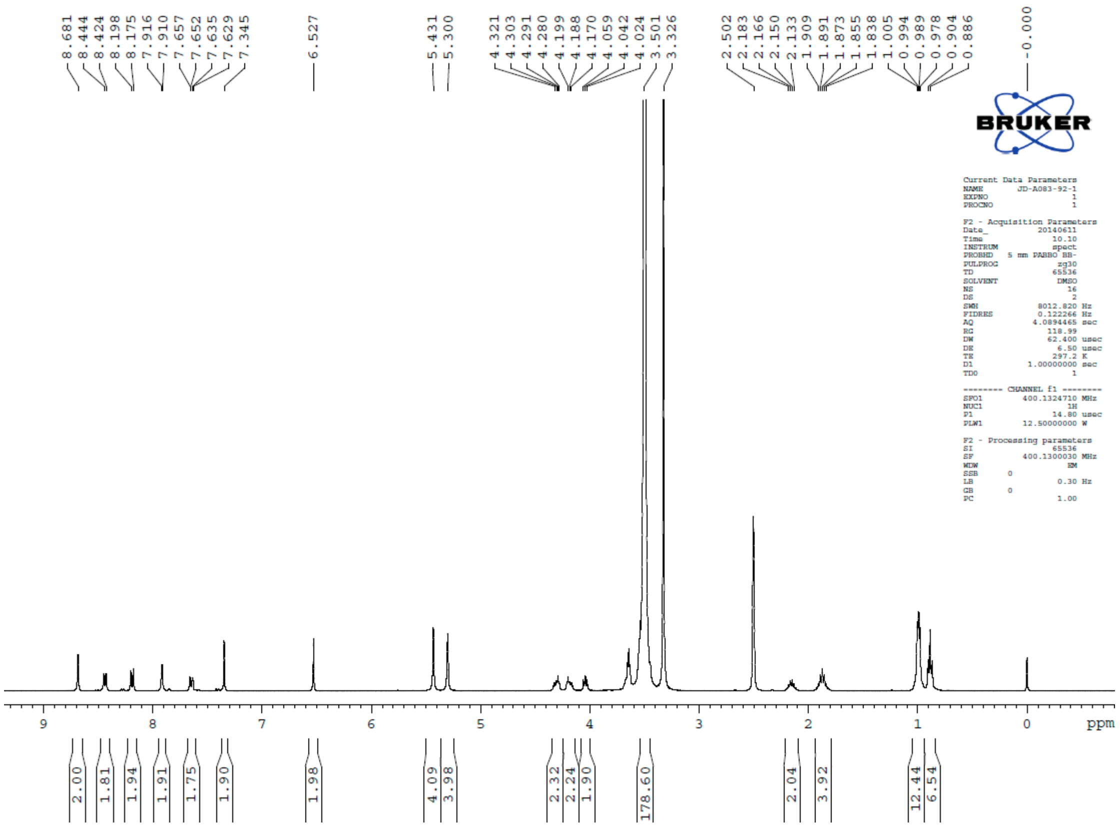

Figure S15. ^{1}H-NMR of compound **4d**.

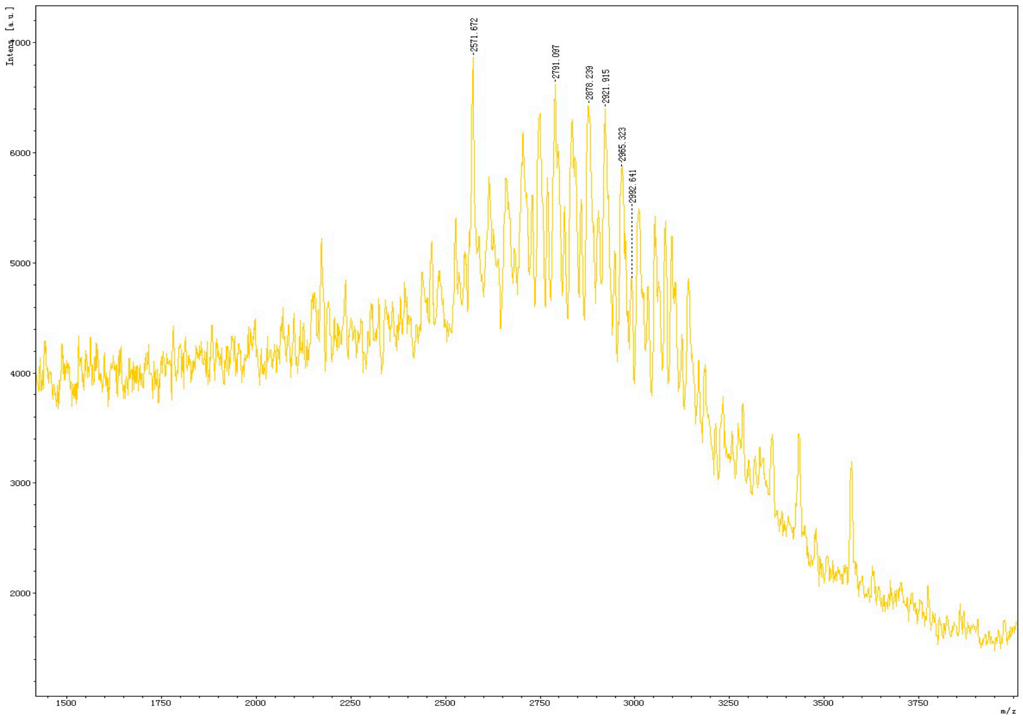

Figure S16. MALDI-TOF of compound **4d**.

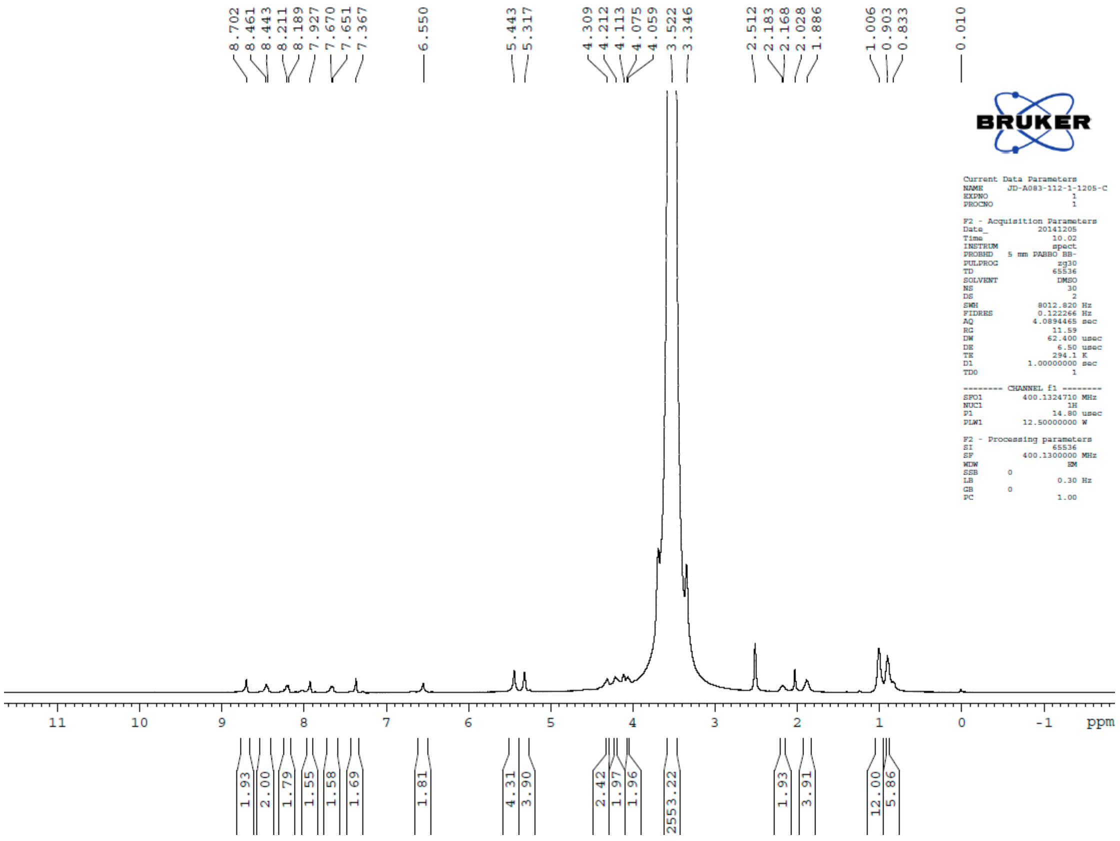

Figure S17. [1] H-NMR of compound **4e**.

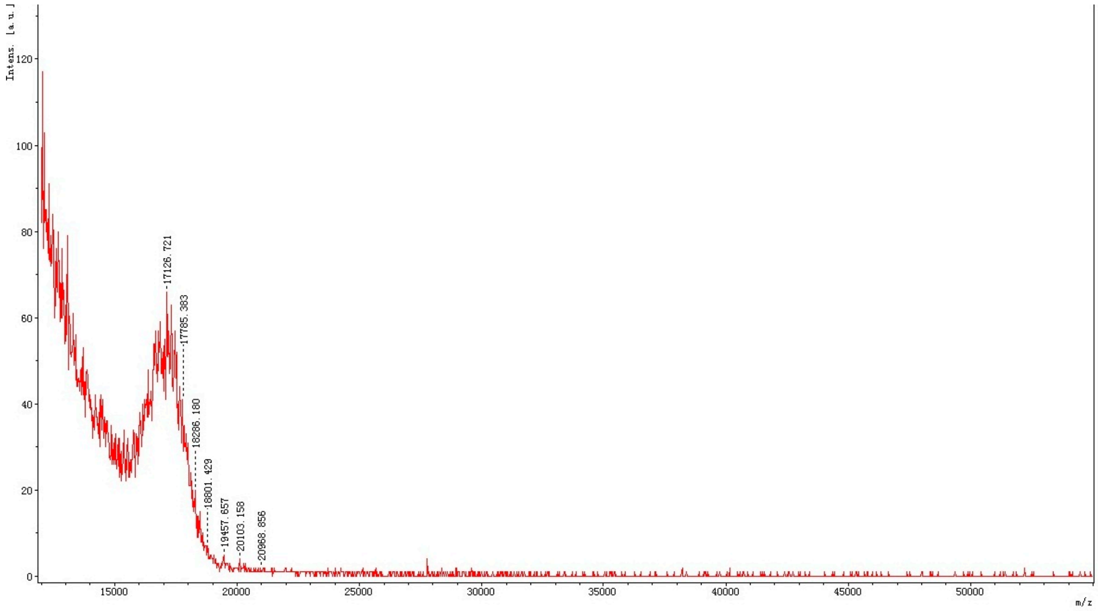

Figure S18. MALDI-TOF of compound **4e**.

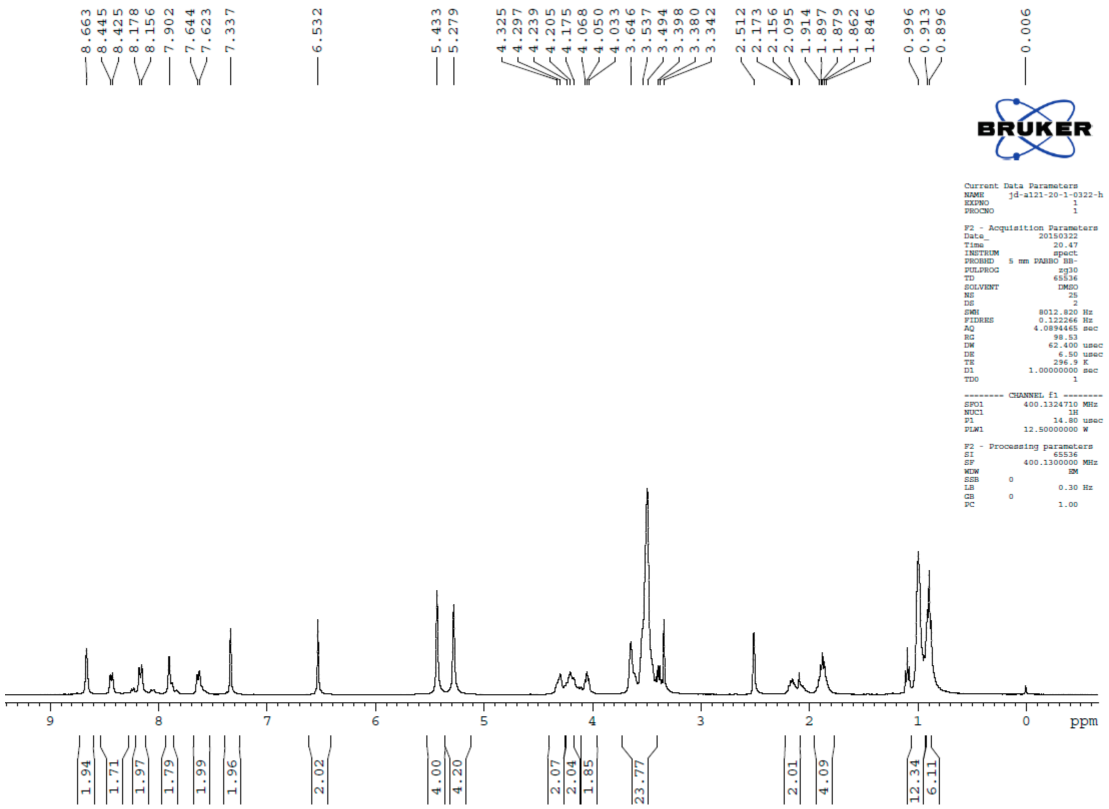

Figure S19. ^{1}H-NMR of compound **4f**.

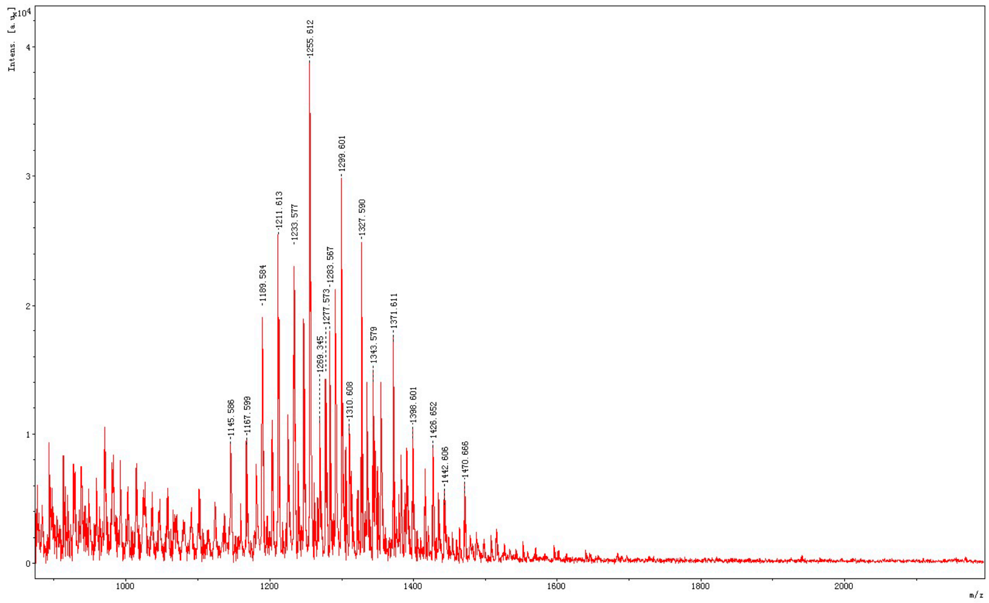

Figure S20. MALDI-TOF of compound **4f**.

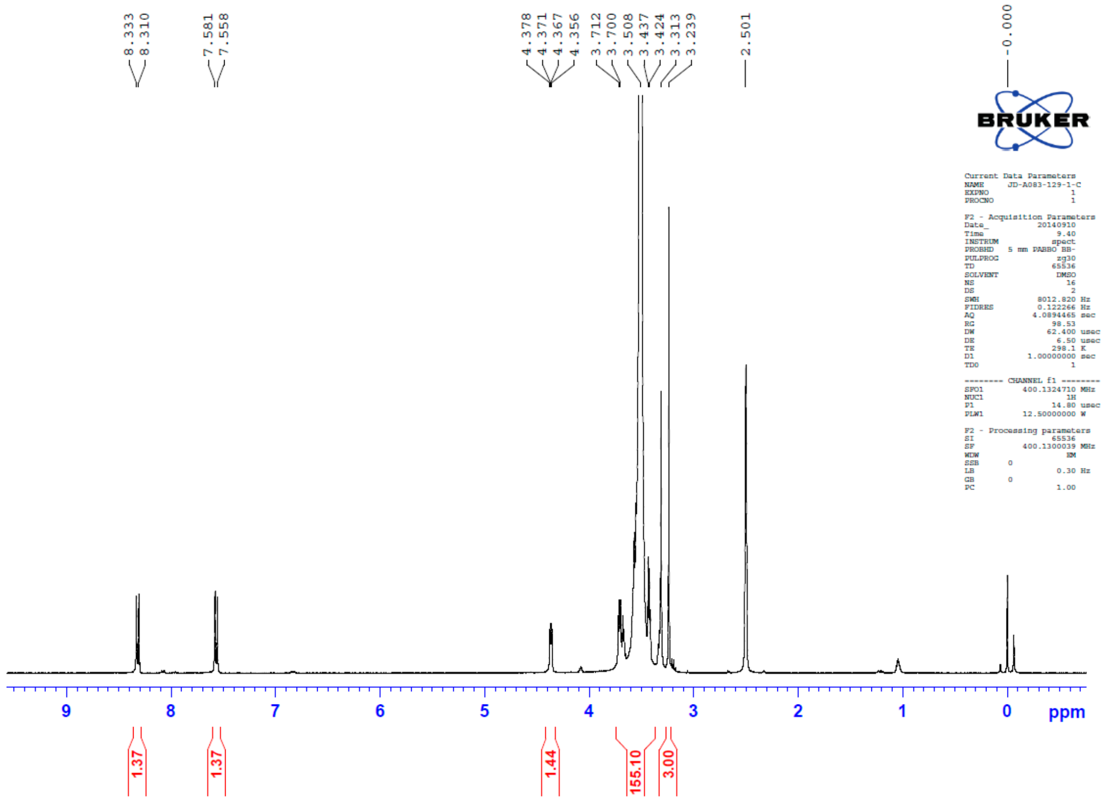

Figure S21. ^{1}H-NMR of compound **1b**.

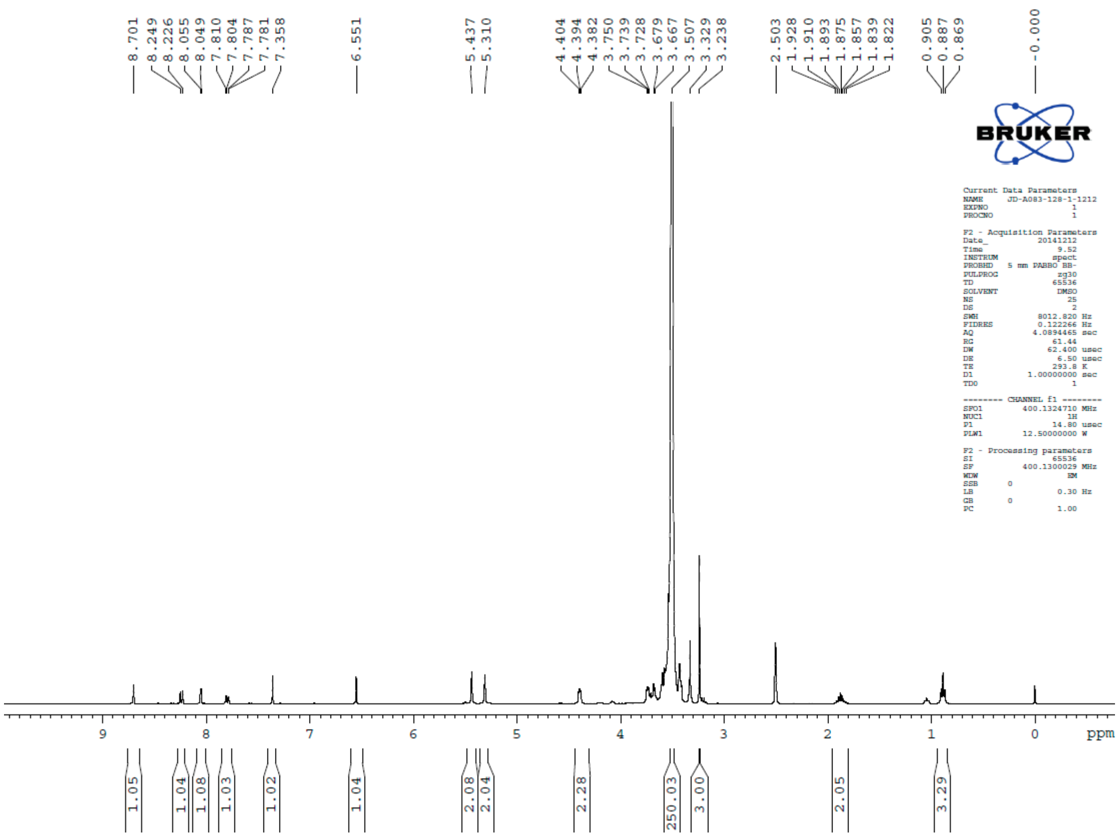

Figure S22. ^{1}H-NMR of compound **5**.

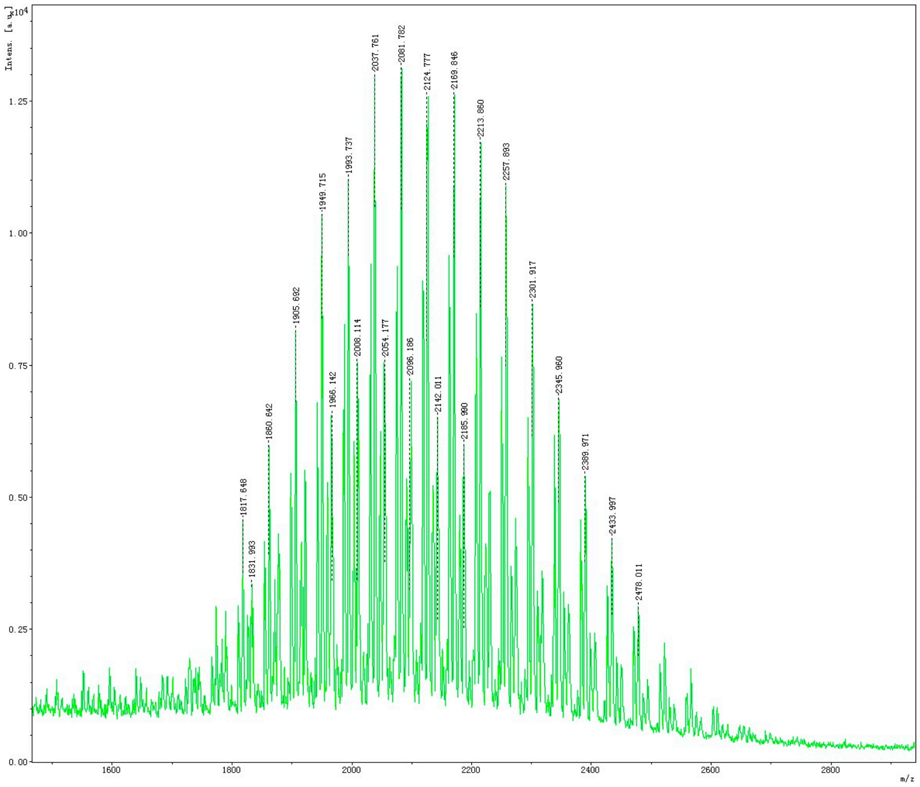

Figure S23. MALDI-TOF of compound **5**.

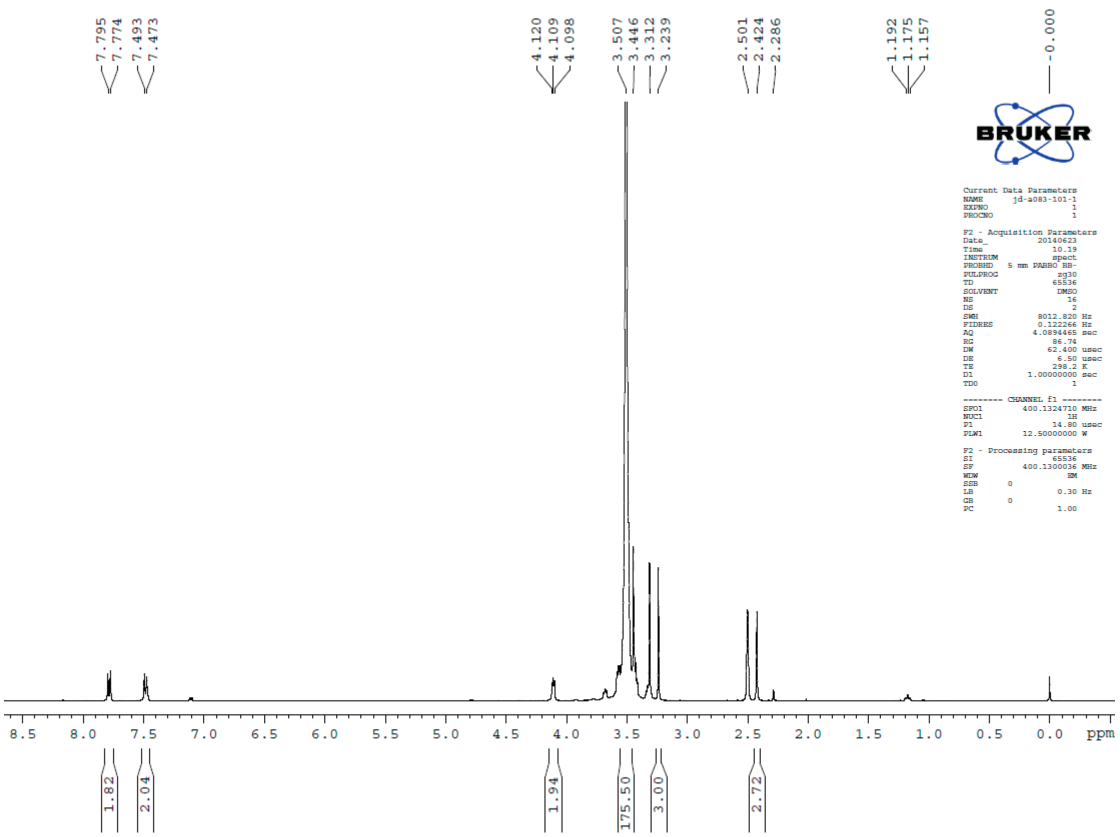

Figure S24. ^{1}H-NMR of compound **7**.

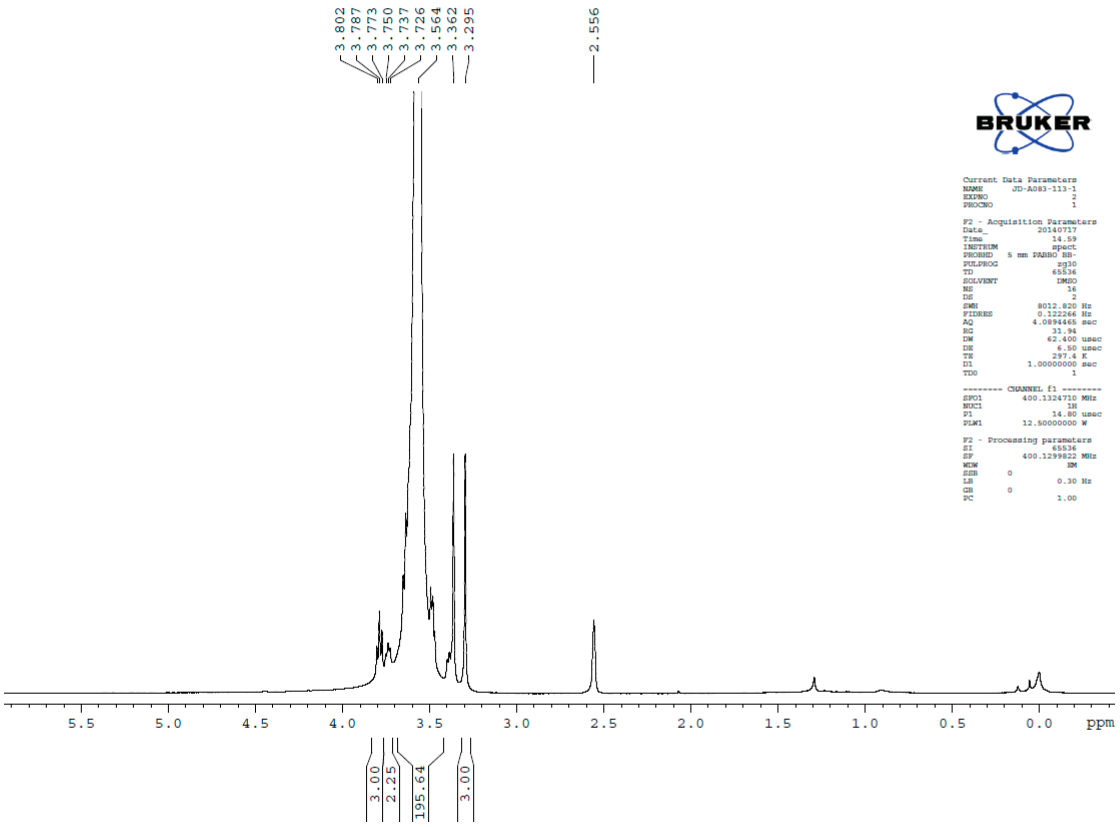

Figure S25. ^{1}H-NMR of compound **8**.

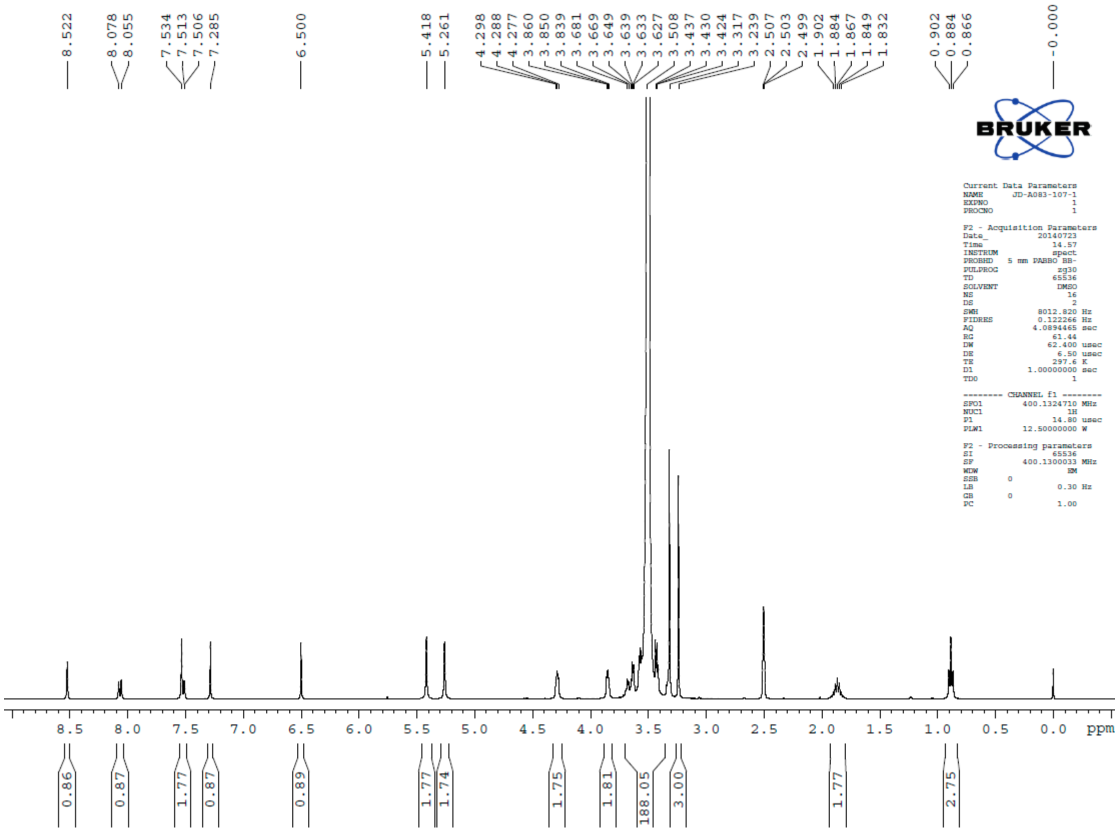

Figure S26. ^{1}H-NMR of compound **6**.

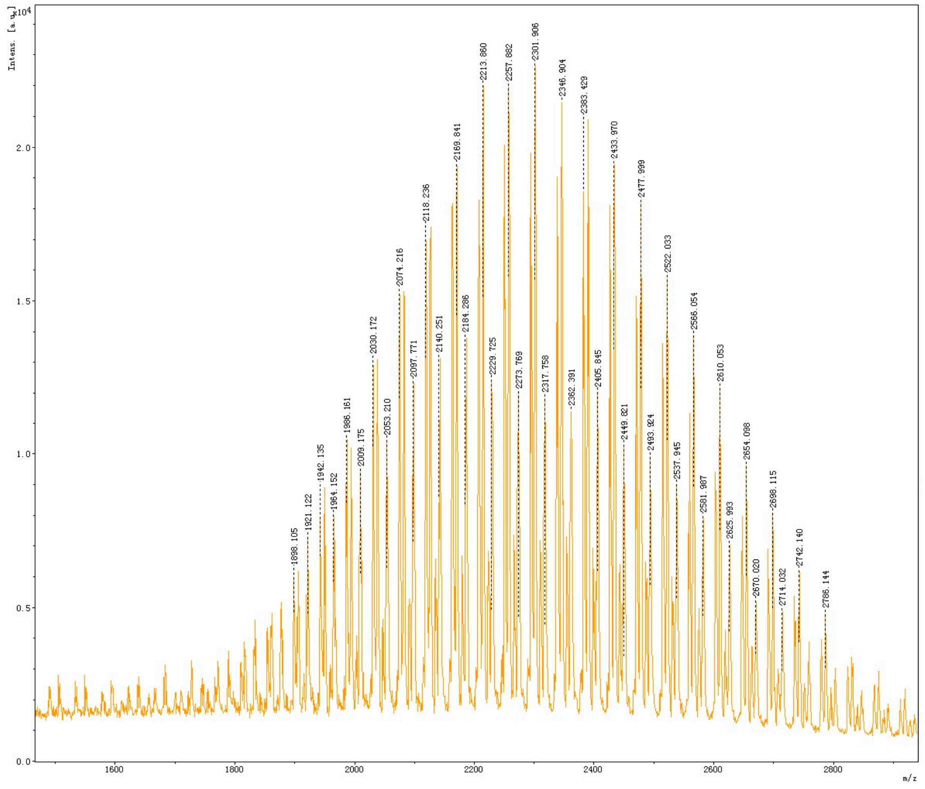

Figure S27. MALDI-TOF of compound **6**.
